# Liver mesenchymal stem cells are superior inhibitors of NK cell functions through differences in their secretome compared to other mesenchymal stem cells

**DOI:** 10.3389/fimmu.2022.952262

**Published:** 2022-09-21

**Authors:** Furkan Yigitbilek, Elif Ozdogan, Nitin Abrol, Walter D. Park, Michael J. Hansen, Surendra Dasari, Mark D. Stegall, Timucin Taner

**Affiliations:** ^1^ Department of Surgery, Mayo Clinic, Rochester, MN, United States; ^2^ Department of Immunology, Mayo Clinic, Rochester, MN, United States; ^3^ Department of Quantitative Health Sciences, Mayo Clinic, Rochester, MN, United States

**Keywords:** mesenchymal stem cells, tolerance, immunomodulation, immune regulation, liver transplant

## Abstract

Liver-resident mesenchymal stem cells (L-MSCs) are superior inhibitors of alloreactive T cell responses compared to their counterparts from bone marrow (BM-MSCs) or adipose tissue (A-MSCs), suggesting a role in liver’s overall tolerogenic microenvironment. Whether L-MSCs also impact NK cell functions differently than other MSCs is not known. We generated and characterized L-MSCs, A-MSCs and BM-MSCs from human tissues. The mass spectrometry analysis demonstrated that L-MSC secretome is uniquely different than that of A-MSC/BM-MSC, with enriched protein sets involved in IFNγ responses and signaling. When co-cultured with primary human NK cells, L-MSCs but not other MSCs, decreased surface expression of activating receptors NKp44 and NKG2D. L-MSCs also decreased IFNγ secretion by IL-2-stimulated NK cells more effectively than other MSCs. Cytolytic function of NK cells were reduced significantly when co-cultured with L-MSCs, whereas A-MSCs or BM-MSCs did not have a major impact. Mechanistic studies showed that the L-MSC-mediated reduction in NK cell cytotoxicity is not through changes in secretion of the cytotoxic proteins Perforin, Granzyme A or B, but through increased production of HLA-C1 found in L-MSC secretome that inhibits NK cells by stimulating their inhibitory receptor KIRDL2/3. L-MSCs are more potent inhibitors of NK cell functions than A-MSC or BM-MSC. Combined with their T cell inhibitory features, these results suggest L-MSCs contribute to the tolerogenic liver microenvironment and liver-induced systemic tolerance often observed after liver transplantation.

## Introduction

The adult liver hosts many cells of the immune system that are components of both the adaptive and innate immune responses. Due to its unique location at the crossroads of endotoxin-rich portal circulation and the systemic circulation, the liver microenvironment favors a more tolerogenic response to most stimuli at steady state (1). Interestingly, the liver-induced immune hyporesponsiveness remains in effect after liver transplantation, evidenced by the overall reduced alloresponses compared to transplants of other organs (2–4). Furthermore, liver appears to have an immunoprotective effect on simultaneously transplanted other organs from the same donor. This phenomenon stays active for a long time and is manifested through both the passive and active processes that decrease overall inflammation in the simultaneously transplanted organs (5–8).

Mesenchymal stem/stromal cells (MSCs) are multipotent cells characterized by self-renewal and multilineage differentiation, which have been extensively investigated since first identified within the bone marrow (9–11). They can be isolated from many tissues including the placenta, neonatal umbilical cord, bone marrow, and adipose tissue (12–17). Although noted initially for their roles in tissue repair and regeneration, studies have demonstrated that the MSCs possess robust immunomodulatory properties through mainly the paracrine factors they produce (18–20). The immunomodulatory properties appear to be influenced by the source of origin, as well as the *in vitro* culture conditions (21). In addition, ontogenetic factors may alter these properties, as MSCs isolated from fetal tissues, especially the fetal liver, have longer-lasting immunomodulatory and immunosuppressive effects (22–25).

Guided by the similarities between the immunomodulatory functions of MSCs and the liver allografts’ tolerogenic properties, we have successfully isolated MSCs from the liver to characterize their properties further (26). We showed that human liver-derived mesenchymal stem/stromal cells (L-MSCs) inhibit alloreactive T cell proliferation, reduce the frequency of IFNγ-producing alloreactive T cells, and have upregulated expression of genes associated with T cell modulation. All these effects of L-MSCs were greater than those of MSCs derived from human bone marrow (BM-MSC) or human adipose tissue (A-MSC) and did not require cell-to-cell contact.

MSCs isolated from bone marrow (27, 28) and fetal liver tissue (22) have also been reported to inhibit the cytolytic functions of NK cells and their cytokine secretion. NK cell responses are an important innate component of transplant rejection (29, 30). In order the understand whether L-MSCs have a different impact on NK phenotype and function, here, we studied the influence of human L-MSC, A-MSC, and BM-MSC on human NK cells by investigating the differences in the MSC secretome and the role of differentially expressed proteins on the functional differences observed in NK cells.

## Material and methods

### Isolation of MSCs from different human tissues

The study was approved by the Mayo Clinic Institutional Review Board. Approximately 1 cm^3^ biopsy of liver tissue was obtained from six brain dead liver donors to isolate L-MSCs. The bone marrow and the subcutaneous abdominal wall adipose tissue were obtained from living kidney donors at the time of organ procurement, as described previously (26). Tissues were processed within 8 hours of procurement. Liver and adipose tissues were minced in a Petri dish and mixed with 0.075% Collagenase IV (STEMCELL Technologies, Cambridge, MA) in Dulbecco’s phosphate-buffered saline for 2 hours. Enzyme action was stopped by adding MSC media made of aMEM (Life Technologies, Grand Island, NY), Glutamax (Life Technologies, Grand Island, NY), Human Platelet Lysate Gold (Mill Creek Life Sciences, Rochester, MN). Digested tissue was filtered twice and centrifuged, then resuspended in fresh MSC media and plated in a 150 cm^2^ flask. Nonadherent cells were removed the next day, and media was changed every three days thereafter. Cultures were maintained at 37°C, 5% CO2, in a humidified incubator. Cells were allowed to proliferate for approximately ten days before being passaged for the first time, and then the cells were passaged when the confluence was between 80% and 90%. TrypLE Select (Life Technologies, Grand Island, NY)) was used to dissociate them for the passaging process. All the cells were frozen down at passages 3 and 4 for further usage. A schematic representation of the experimental protocol can be found in **Supplementary Figure 1**.

### Characterization of MSCs

All the MSCs used in the current study were characterized in our previous study (26). Briefly, characterization of MSCs was done according to The International Society for Cellular Therapy guidelines (9). Cultured MSCs demonstrated plastic-adherent characteristics and a spindle-shaped morphology. The differentiation ability of MSCs into adipocyte, osteocyte, and chondrocyte lineages was assessed using the Human MSC Functional Identification Kit (R&D Systems, Minneapolis, MN), following the manufacturer’s recommended protocol. After the second passage, the phenotype characterization of MSCs was done using the fluorochrome-conjugated monoclonal antibodies of canonical MSC markers by flow cytometry (CD44, CD45, CD90 and CD105 from Beckman Coulter, Brea, CA; CD73 from BD Biosciences, Franklin Lakes, NJ). HLA typing of the cells was done using a DNA-based sequence-specific primer method using LABType (One Lambda, Canoga Park, CA), as described previously (31). Data were analyzed using Kaluza software (Beckman Coulter, Chaska, MN).

### Mass spectrometry proteomic analysis

All MSCs were cultured simultaneously and under the same conditions, until 80-90% confluence. Supernatants were then collected, cell debris removed by centrifugation at 400g for 20 min and stored at -80°C. We utilized a previously published peptide intensity-based label-free quantification method to identify differentially expressed proteins between experimental groups (32). In brief, proteins extracted from the sample matrix were denatured *via* heat and sonication. Cysteine disulfide bridges in the denatured proteins were reduced and alkylated using iodoacetamide. Reduced and alkylated proteins were digested into peptides by incubating them at room temperature overnight with the porcine trypsin enzyme. Digestion was halted *via* acidification, and peptide digests were cleaned for mass spectrometry analysis. Digests were loaded onto a Dionex-QExactive liquid chromatography-tandem mass spectrometry (LC-MS/MS) system for analysis. The mass spectrometer was configured to perform data-dependent acquisition. The resulting raw data were processed using MaxQuant software to identify peptide sequences and infer protein identifications (33). The MaxQuant software was configured to search the human protein sequence database by assuming trypsin as digestion enzyme, QExactive as a mass spectrometer, and the following as variable posttranslational modifications: carbamidomethylation of cysteine, oxidation of proline, and n-terminal pyroglutamic acid formation. The software was configured to add reversed protein sequences to the search database for estimating peptide and protein false discovery rates (FDRs). The resulting protein and peptide identifications were filtered at 1% FDR. Precursor intensities of filtered proteins were quantile normalized to account for technical variation.

### Bioinformatics of secretome analysis

Normalized protein intensities were independently compared across experimental groups using a Gaussian distribution-based generalized linear model (GLM). This model was contrasted with a GLM model configured with randomized sample order as expected background variation in protein intensities. A chi-square test was then utilized to assess the statistical significance of a protein’s intensity difference between experimental groups. The resulting P values were FDR corrected using the Benjamini-Hochberg method, and significantly differentially expressed proteins (FDR ≤ 0.05) were considered for further analysis. Principal component analysis and clustered heatmap analysis were done using Partek Bioinformatics Software to investigate differences between the three MSC sources.

Gene ontology analysis of proteins was done using GSEA software version 4.1.0, which uses gene sets from the Molecular Signatures Database (MsigDB v7.4). We used the C5 gene ontology (GO) collection with 1000 permutations for the current study. The minimum and maximum criteria for selecting gene sets from the collection were 15 and 500 genes, respectively.

The list of all expressed proteins in the L-MSCs compared to nL-MSCs was uploaded with gene identifiers, corresponding fold change, and P value into the Ingenuity Pathway Analysis (IPA) software. The core analysis function was used to interpret the data, including biological processes and upstream analysis.

### Natural killer cell isolation

Primary NK cells were isolated from apheresis cones obtained from blood donors using the RosetteSep™ Human NK Cell Enrichment Cocktail (Stem Cell Technologies, Cambridge, MA) designed to isolate NK cells from whole blood by negative selection. The blood product was diluted 1:1 with sterile PBS and layered over Ficoll–paque^®^ plus (GE Healthcare Life Science, Uppsala, Sweden) at room temperature, then spun at 400 g for 30 min with no brake at room temperature to obtain peripheral blood mononuclear cells (PBMCs). After that, some of the PBMCs were saved to use in flow cytometry analyses, and the remaining were mixed with 100-fold excess red blood cells from the same waste product and centrifuged for 5 min at 400 g. NK cell separation antibody mixture was added (1 µL per million PBMCs) and mixed gently by swirling every 5 minutes for 20 minutes at room temperature to isolate NK cells. Following 20 minutes, the mixture was layered over Ficoll–paque^®^ plus and spun at 400 g for 30 min with no brake at room temperature. The resulting NK cell layer was then collected and washed three times with PBS. A small sample was then taken for flow cytometry-based purity analysis, and the remaining cells were mixed to minimize the donor-related differences between the cells and placed in culture media (RPMI) (Life Technologies, Carlsbad, CA) supplemented with penicillin and streptomycin (Pen-Strep; 10,000 U/ml), 200 mM L-glutamine, 100 mM sodium pyruvate, and 0.01 mM MEM non-essential amino acids bought from Corning, Manassas, VA, and 10% FBS (Thermo Fisher Scientific, Waltham, MA) with and without 100 U/ml recombinant human (rh)IL-2 (Peprotech, Cranbury, NJ) overnight.

### Co-culture of MSCs and NK cells

Forty thousand L-MSCs, BM-MSCs, and A-MSCs were plated separately in 75cm^2^ flasks in 10 ml MSC-medium and allowed to adhere to the plate for 24hr. After overnight adherence, the media was aspirated, and 4x10^6^ primary NK cells were added in 20mL medium (10mL NK medium and 10 mL MSC medium) with stimulation of 100U/mL IL-2 to maintain the 10:1 NK cell/MSC ratio. For controls, NK cells were cultured in 10 ml MSC-medium and 10 ml NK cell-medium with or without IL-2. In some experiments, blocking antibodies to KIR3DL1 (R&D Systems, Minneapolis, MN) (5 µg/mL) and KIR2DL2/3 (ProteoGenix, Schiltigheim, France) (10 µg/mL) were added at the beginning. After 3 days of culturing at 37°C, 5% CO2, in a humidified incubator, NK cells were harvested from co-cultures by gentle pipetting, washed and their survival assessed. The NK cell survival was estimated as follows: [(Number of harvested cells/Number of plated cells) x 100]. Notably, NK cells did not contain any significant contamination of MSCs, which grow as adherent cells. NK cell viability was as assessed by trypan blue staining as well as annexin V/Propidium Iodide dual staining (ThermoFisher Scientific, Waltham, MA) using flow cytometry.

For the experiments testing the role of cell-to-cell contact, NK cells were cultured with the MSC culture supernatants for 3 days, without the presence of any MSCs. MSC supernatants were obtained as described above, after 80-90% confluence was reached in cell cultures. After collection of the supernatants, cell debris was removed by centrifugation at 400g for 20 min and supernatants stored at -80°C until they were used.

### Flow cytometry profiling

Following co-culture with MSCs, NK cells were collected, washed and the natural cytotoxicity receptors (NCRs) expressed by NK cells were analyzed by flow cytometry using the following human monoclonal antibodies: CD3 (FITC), NKp30 (APC), NKp44 (PE), NKp46 (PerCp-Cy5.5), NKG2D (Apc-Cy7) from Biolegend, San Diego, CA; CD56 (Pe-Cy7), CD16 (VF-450), and Ghost Dye (V-510) from Tonbo Biosciences, San Diego, CA. The cells were stained for 30 minutes at 4°C. Samples were analyzed using the Beckman Coulter Gallios 3-laser, 10-color flow cytometer, and FlowJo software.

### Chromium-51 release cytolytic assays

The cytolytic activity of NK cells was determined using a 4-hour Chromium-51 (Cr51) release assay. The K562 cell lines were used as the target cell. Target cell lines (American Type Culture Collection, Rockville, MD) were maintained in RPMI with 10% FBS and Pen-Strep, and pulsed with Cr51 (Perkin Elmer, Boston, MA) for a minimum of 1 hr and then washed with PBS to remove excess Cr51. Primary NK cells were plated in a 96-well plate at the indicated dilutions and then incubated with Cr51-labeled target cells for 4 hours to allow interaction and subsequent cytotoxicity. The plate was centrifuged, the medium supernatant removed onto a Luma-Plate 96 (Perkin Elmer, Boston, MA) and allowed to dry overnight before sealing with Top-Seal A Plus (Perkin Elmer, Boston, MA) and read on a Top Count NXT Microplate Scintillation and Luminescence Counter. All experimental groups were tested as triplicates. All values shown are relative to maximum lysis of target cells (MAX), as defined by Cr51 release in 0.5% Triton X-100 (Sigma-Aldrich, St. Louis, MO) lysis solution, and spontaneous lysis (MIN), as defined by Cr51 release in NK-cell media in the absence of cells. Specific Cr51 release was calculated for each sample by the following equation: Percent Specific Lysis: [(Experimental Release – Spontaneous Release)/(Maximum Release – Spontaneous Release)] x 100.

### Enzyme-linked immunosorbent assay

The supernatants from the cell cultures were collected, centrifuged and stored at -80 °C until used for enzyme-linked immunosorbent assay (ELISA). The protein levels of interferon-γ (IFNγ), tumor necrosis factor-α (TNFα), granzyme A, granzyme B (R&D Systems, Minneapolis, MN), and perforin (Abcam, France) in the supernatant were determined using ELISA techniques according to the manufacturer’s instruction.

### Statistical analyses

Normally distributed data were represented as mean ± standard deviation, and non-normal data as median and interquartile range. Comparisons among groups were performed using the two-sample t-test with a 5% type-I error. All data were considered significant if P ≤0.05. Statistical analysis was accomplished using JMP software (SAS Institute, Cary, NC, United States).

## Results

### Characterization of MSCs from different tissue sources

All MSCs isolated from the human liver (L-MSC, n=6), adipose tissue (A-MSC, n=3), and bone marrow (BM-MSC, n=3) were cultured in MSC media. MSCs used in the current study were characterized previously according to The International Society for Cellular Therapy guidelines. MSC characterization and differentiation were repeated for the current study. (**Supplementary Figure 2**). Briefly, MSCs from all sources were adherent to plastic, and positive for canonical markers CD44, CD73, CD90 and CD105, while they did not express CD45 or HLA class-II. All cell lines differentiated into osteoblasts, chondrocytes and adipocytes in induced differentiation cultures, verifying their trilineage multipotency.

**Figure 1:**
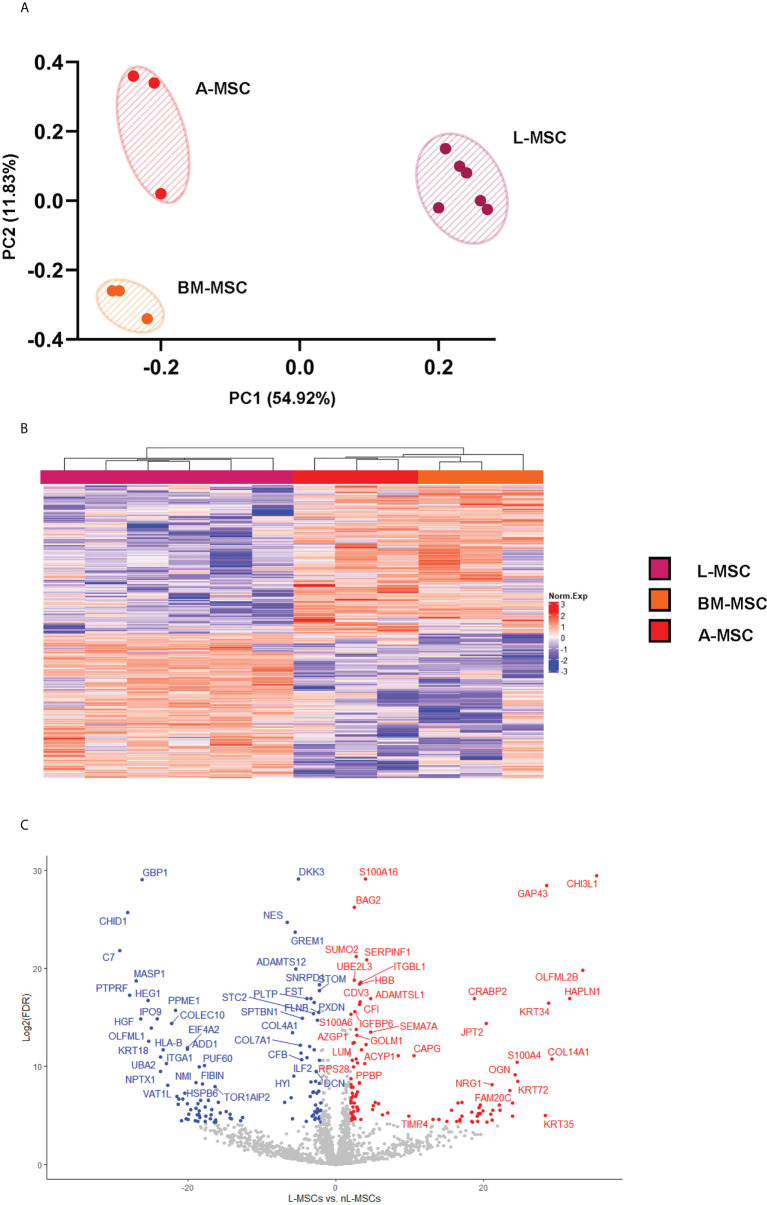
L-MSC secretome is uniquely dissimilar to that of BM-MSCs or A-MSCs. Supernatants from the MSC cultures were collected and subjected to proteomic analysis. **(A)** The PCA demonstrates that the MSC secretomes from different sources cluster together, with intragroup homogenicity and intergroup heterogenicity. **(B)** The heatmap shows that the L-MSC secretome is significantly different that the secretomes of BM-MSC and A-MSC. **(C)** The volcano plot demonstrates the relationship between the magnitude of protein expression change (log2 of fold-change; x-axis) and statistical significance of this change compared to L-MSCs and nL-MSCs. L-MSC, liver mesenchymal stromal cell; BM-MSC, bone marrow mesenchymal stromal cell; A-MSC, adipose mesenchymal stromal cell; nL-MSC, non-liver MSCs.

Secretome analysis of L-MSC (n=6) supernatants was performed based on the protein intensities measured by LC/MS-MS, and the results were compared to those of BM-MSCs and A-MSCs (n=3 each). After normalization, a total of 1792 proteins were identified at different intensities across the three different sources of MSC. To investigate the differences between L-MSCs and their counterparts in more detail, A-MSCs and BM-MSCs were grouped together as non-Liver MSC proteins (nL-MSCs). A total of 582 proteins were considered differentially expressed between the L-MSCs and nL-MSCs with a log-fold change ≥ ± 2 and P value ≤0.05 (**Supplementary Table 1**). Of those, 204 were higher in L-MSC secretome versus the nL-MSC secretome. A principal component analysis of the secretome was performed, and this demonstrated that MSC from each source clustered together, suggesting that the source of MSC had the most influence on the secretome profile (**Figure 1A**). This pattern was further confirmed by the heatmap and the volcano plot (**Figures 1B, C** **)**. Even though there were some differences in protein intensities within each group, these differences were not as significant as those found between different sources of MSCs.

### Protein ontology analysis

**Figure 2:**
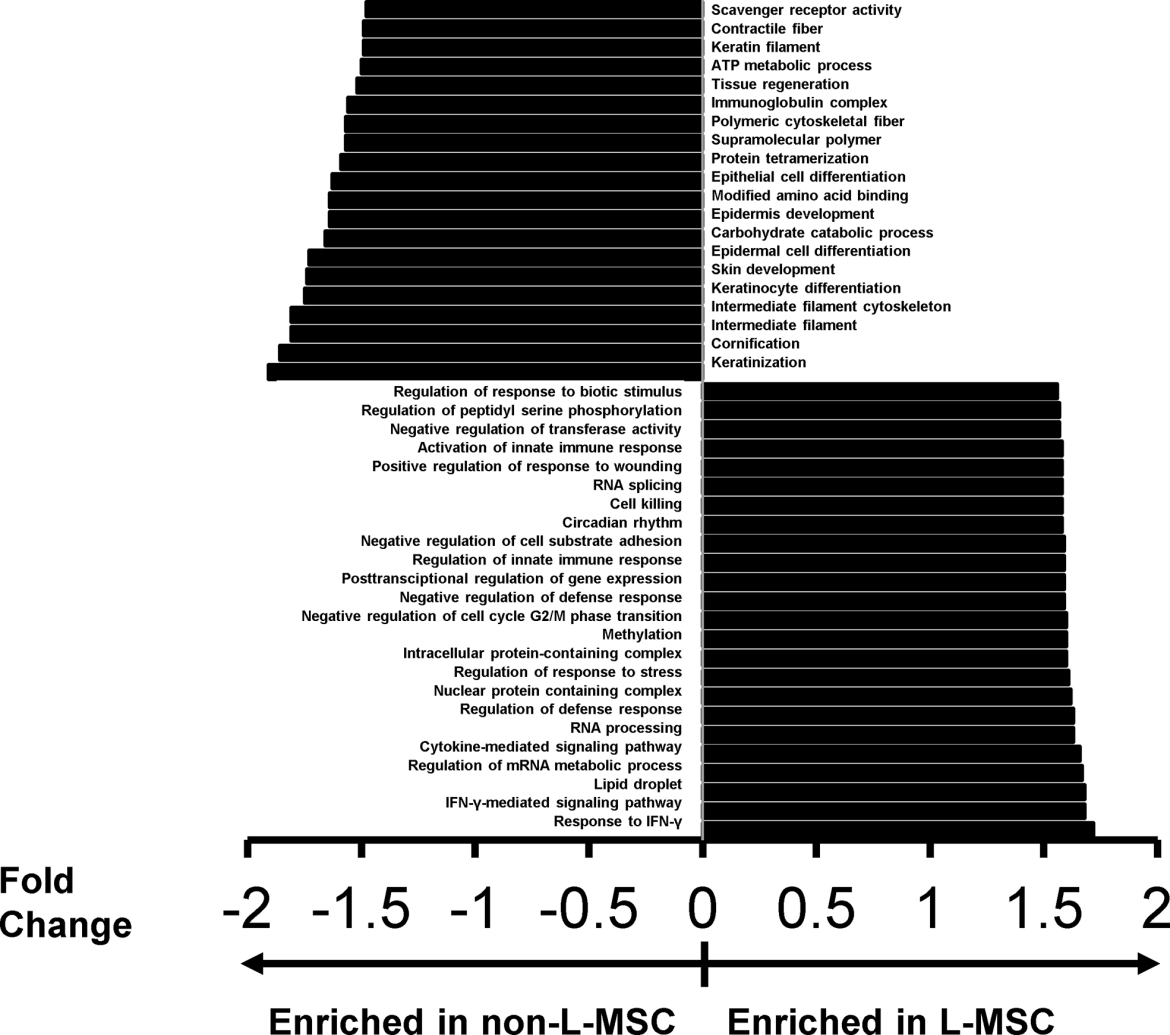
The top protein sets that are differentially expressed (P<0.01) in L-MSC versus non-L-MSC secretome. The secretome data was subjected to protein ontology analysis to investigate the correlations between the intergroup differences and the functional changes observed on the immunomodulatory roles of MSCs from different tissue sources. Protein sets are that are enriched in the secretome of non-L-MSC are demonstrated at the top, and those enriched in the secretome of L-MSC are demonstrated at the bottom.

To better understand the differential expression of the classes of proteins and protein complexes among MSCs derived from different sources, and to correlate these differences with the functional changes observed on the immunomodulatory roles of MSCs, we performed protein ontology analysis. First, a gene set enrichment analysis, a pathway enrichment method that uses protein expression intensity to analyze the main enriched ontology terms categorized based on molecular function and biological process, was used to investigate the differences between the secretome of L-MSCs and nL-MSCs. For this, all proteins detected in L-MSC and nL-MSC secretomes were uploaded for an unsupervised analysis. A total of 70 gene/protein sets were found to be enriched (nominal P<0.01) in L-MSC secretome, while 30 gene/protein sets were enriched in nL-MSC secretome. Remarkably, the top biological processes for L-MSCs were those related to response to IFNγ (NES: 1.72) and IFNγ-mediated signaling pathway (NES: 1.68), whereas the top processes for nL-MSC were keratinization (NES: -1.91) and cornification (NES: 1.83). The most enriched protein ontology sets for L-MSC and nL-MSC secretome are presented in **Figure 2**.

To evaluate the correlations between the proteins unique to L-MSC secretome and previously reported cellular mechanisms, we compared our dataset with other gene/protein sets. The expressed proteins found in L-MSCs were highly associated with cellular functions such as motility (P=2.97E-45-1.12E-06; 259 molecules), cell compromise (P=3.13E-43-1.56E-07; 119 molecules), inflammatory response including activation/inhibition of innate immune responses (P=3.13E-43-8.45E-07; 306 molecules), immune cell trafficking (P=1.08E-29-8.45E-07; 149 molecules), cell death and survival (P=7.24E-29-7.28E-07; 292 molecules), expression of RNA (P=7.94E-14; 268 molecules), and invasion of tumor cell lines (P=4.59E-20; 151 molecules).

### NK cell – MSC co-cultures and survival of NK cells

**Figure 3:**
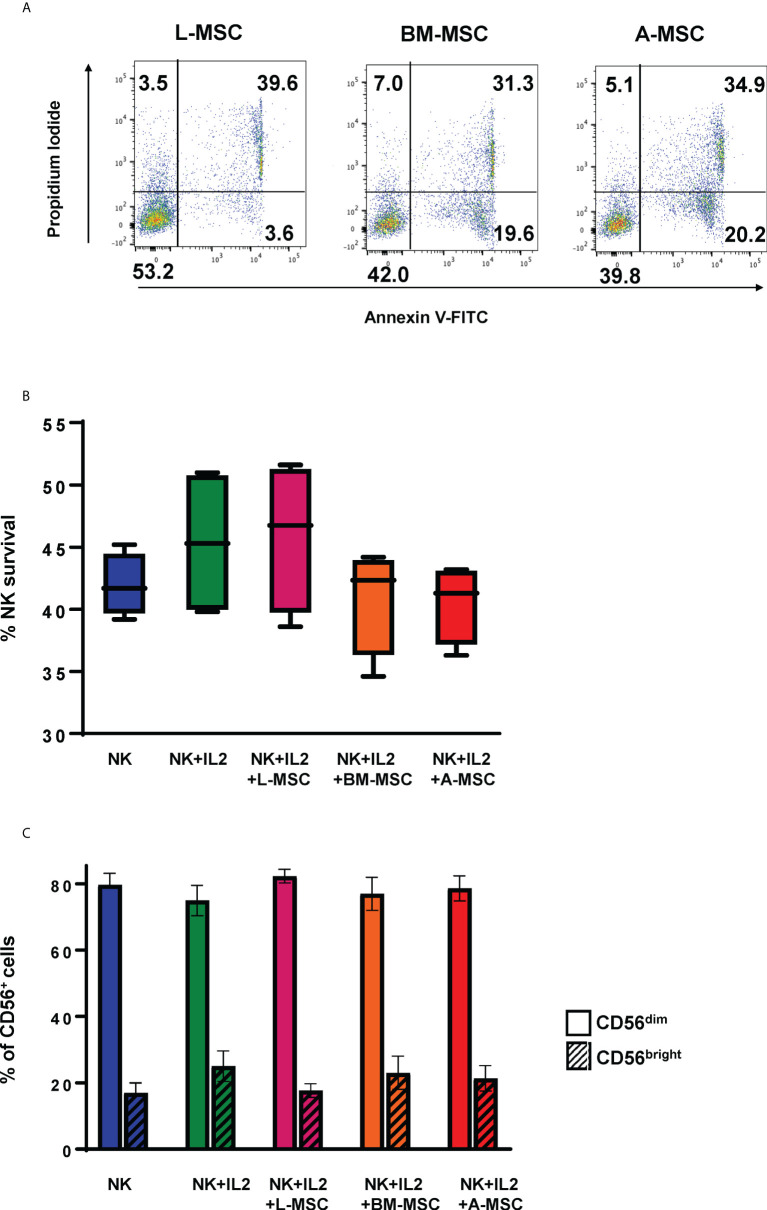
Survival of the NK cells and their subset differentiation after co-culturing with MSC. NK cells were co-cultured with MSC for 3 days (with 100 U/ml rhIL-2), and their survival analyzed by both Annexin V/Propidium Iodide dual staining **(A)** and trypan blue dye exclusion **(B)**. No difference was observed in NK cell survival between the different MSC co-cultures. Data presented in panel A is representative of 4 independent experiments. Data presented in panel B represents mean percent viable NK cells ± SD of 5 independent experiments, analyzed by Student’s pair t-test. **(C)** The phenotype of NK cells was assessed by flow cytometry after co-cultures with MSCs for 3 days. Percentages of CD56^bright^ and CD56^dim^ are shown from 3 different experiments with mean values ± SD. Data were analyzed using Student’s pair t-test. L-MSC, liver mesenchymal stromal cell; BM-MSC, bone marrow mesenchymal stromal cell; A-MSC, adipose mesenchymal stromal cell; NK, natural killer.

Given the significant differences found in the protein sets involved in activation/inhibition of innate immune responses between the L-MSCs and nL-MSCs, next, we focused on NK cell phenotype and functions when cultured with different types of MSCs. Freshly isolated NK cells were co-cultured with MSCs for three days. Throughout the 3-day coculture period, no significant cell death was observed among MSCs which stayed adherent to the cell plate (data not shown). NK cell viability at the end of the culture period was assessed with trypan blue exclusion, as well as annexin V/Propidium Iodide dual staining (**Figure 3A**). The mean survival of NK cells was 45.9% ± 11.2 for NK cells supplemented with IL-2 and 43.1% ± 4.1 without IL-2. Overall, co-culture with MSCs did not change the survival with the mean survival of 50.3% ± 11.1, 44.3% ± 9.4, 39% ± 15.6 for L-MSCs, BM-MSCs, and A-MSCs, respectively **(** **Figure 3B**).

### NK Cell phenotype and subset changes in MSC co-cultures

NK subsets have different biological functions and roles in different disease processes. To assess the changes in NK cell subsets in MSC co-cultures, phenotypic analyses of NK cells were performed at the end of the co-culture period. As expected, exposure to IL-2 increased the percentage of CD56^bright^ NK cells from 16.3% ± 3.8 to 22.8% ± 7.5 (P=0.72). Co-culturing the NK cells with MSCs reversed this shift with CD56^bright^ frequencies of 12.4% ± 7.8, 13.7% ± 11.7, 12.8% ± 8.0 for L-MSCs, BM-MSCs, and A-MSCs, respectively (**Figure 3C**, **Supplementary Figure 3**).

**Figure 4:**
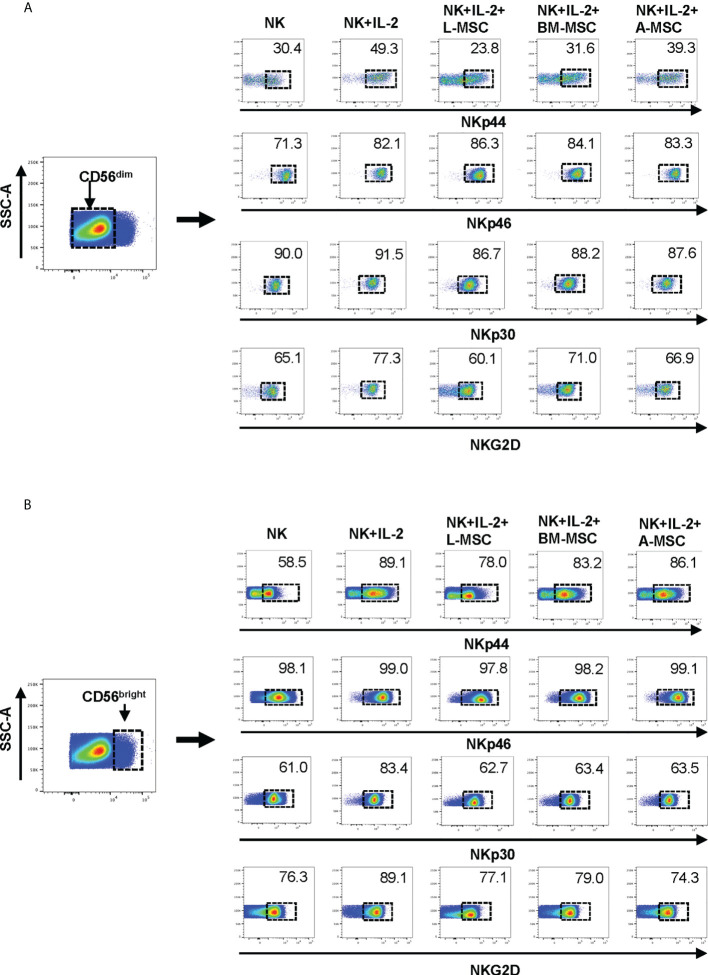
Expression of the NK receptors on CD56^dim^ and CD56^bright^ NK cells after co-culturing with MSC. After co-culturing with MSC for 3 days (with 100 U/ml rhIL-2), NK cells were harvested, and surface expression of NKp30, NKp44, NKp46 and NKG2D was examined by flow cytometry, after gating for the CD56^dim^ and CD56^bright^ populations. **(A)** A representative dot plot of 4 independent experiments shows that among the CD56^dim^ NK cells, the percentage of NKp44-positive and NKp46-positive cells increase after stimulation with IL-2. L-MSC decreases the percentage of both NKp44-positive and NKG2D-positive NK cells. **(B)** A representative dot plot of 4 independent experiments shows that the percentage of NKp30-positive cell among the CD56^bright^ NK population increases after stimulation with IL-2, and this is reversed by all three types of MSC. The mean percent-positive values ± SD of the cumulative data using each of the MSC lines are presented in **Table 1**.

Next, we investigated NK receptor expression among the CD56^dim^ and CD56^bright^ subsets after IL-2 stimulation in the absence or presence of MSCs. Stimulation of NK cells with IL-2 led to an increase in the frequency of CD56^dim^ NK cells expressing the activating receptors NKp44 (44.4% ± 9.6 vs. 7.1% ± 6; P<0.005) and NKp46 (83.5% ± 6.1 vs. 66.9% ± 8.6; P<0.05, but no significant difference was observed in the percentage of NKp30 or NKG2D-positve cells (**Figure 4A**, **Table 1** **)**. When cultured with L-MSCs, the frequency of the NKp44-positive subset was decreased to 19.2% ± 11.1 (P<0.05) on CD56^dim^ NK cells. Although co-culturing with A-MSCs and BM-MSCs also decreased the percentage of NKp44-positive CD56^dim^ NK cells, these were not statistically significant. L-MSCs also reduced the percentage of NKG2D-positive CD56^dim^ NK cells (61.4% ± 9.7 vs. 76.8% ± 4; P<0.05), while A-MSCs or BM-MSCs did not have a significant impact. None of the MSCs had a significant impact on the percentage of NKp30-positive NK cells.

**Table 1:** Surface expression of NK receptors on CD56^dim^ NK cells.

	CD16	NKp30	NKp44	NKp46	NKG2D
**NK Cell**	94.7 ± 1.4	88 ± 8.9	27.1 ± 6	66.9 ± 8.6	63.8 ± 9
**NK Cell + IL2 **	83.1 ± 8.9	88.5 ± 10.8	44.4 ± 9.6**	83.5 ± 6.1*	76.8 ± 4
**NK Cell + IL2 + L-MSC**	82.4 ± 9.8*	84.9 ± 13.4	19.2 ± 11.1^#^	85.7 ± 5.1^*^	61.4 ± 9.7^#^
**NK Cell + IL2 + BM-MSC**	80.7 ± 5.2*	83.4 ± 9.6	27.4 ± 14.1*	84.1 ± 7.9*	68.4 ± 4.8
**NK Cell + IL2 + A-MSC**	85.3 ± 4.2	85.6 ± 13	31.9 ± 10*	85.2 ± 5.5*	65.3 ± 14

NK cells were cocultured for 3 days, in either absence or presence of different MSCs at 10:1 NK : MSC ratio. The expression levels of CD16, NKp30, NKp44, NKp46, NKG2D antigens on NK cells were examined by flow cytometry using the monoclonal antibodies after selecting the population of CD56^dim^. The data are presented as the means ± SD of the percentages of NK cells that expressed a given marker from 4 independent experiments. *p < 0.05; **p < 0.005; vs NK cells; ^#^p < 0.05vs NK cells + IL2. Paired Student’s t-test was used for the analysis.

Among the CD56^bright^ NK cells, while stimulation with IL-2 increased the frequency of NKp30 expressing cells, all three MSC types reversed this upregulation. Similarly, the IL-2-mediated upregulation of NKG2D was reversed by both the L-MSCs and A-MSCs. None of the MSC types appeared to have an impact on the frequency of NKp44 or NKp46 positive CD56^bright^ NK cells (**Figure 4B**, **Table 2**).

**Table 2:** Surface expression of NK receptors on CD56^bright^ NK cells.

	CD16	NKp30	NKp44	NKp46	NKG2D
**NK Cell**	25.9 ± 12	59.4 ± 12.6^#^	57.1 ± 4.2	99 ± 0.5	77.2 ± 2.1^#^
**NK Cell + IL2**	26.2 ± 6.7	82.5 ± 9.2	90 ± 7.2 ***	99.1 ± 0.5	88.4 ± 4.1
**NK Cell + IL2 + L-MSC**	38.1 ± 11.5	61.2 ± 10.6^#^	77.2 ± 10.3*	98.8 ± 0.6	77.5 ± 6.8^#^
**NK Cell + IL2 + BM-MSC**	32.8 ± 9.4	61.9 ± 12^#^	84.7 ± 7.1**	99.1 ± 0.4	79.4 ± 3.8
**NK Cell + IL2 + A-MSC**	34.4 ± 7.2	60.7 ± 12.5^#^	86.6 ± 8.3**	99 ± 0.6	75.2 ± 8.2^#^

NK cells were cocultured for 3 days, in either absence or presence of different MSCs at 10:1 NK : MSC ratio. The expression levels of CD16, NKp30, NKp44, NKp46, NKG2D antigens on NK cells were examined by flow cytometry using the monoclonal antibodies after selecting the population of CD56^bright^. The data are presented as the means ± SD of the percentages of NK cells that expressed a given marker from 4 independent experiments. *p < 0.05; **p < 0.005; ***p < 0.005 vs NK cells; ^#^p < 0.05vs NK cells + IL2. Paired Student’s t-test was used for the analysis.

### MSCs modulate cytokine secretion by NK cells

**Figure 5:**
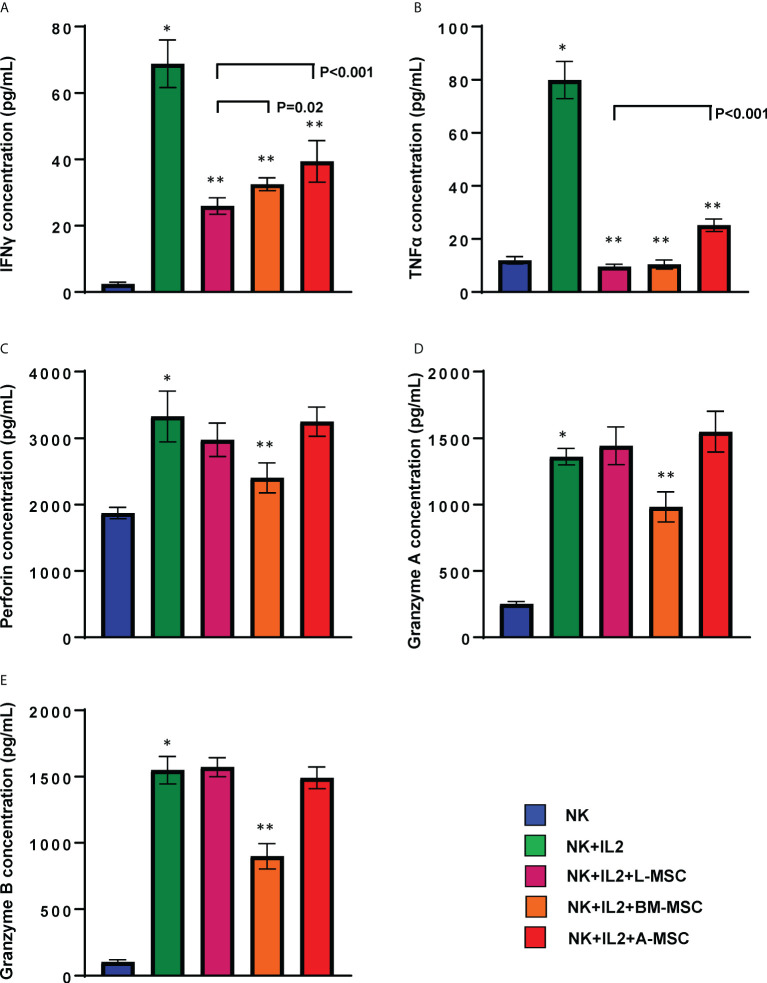
MSCs’ modulation of cytokine secretion by NK cells. Following 3 days of co-culture with IL-2, supernatants were collected, and their IFNγ **(A)**, TNFα **(B)**, Perforin **(C)**, Granzyme A **(D)** and Granzyme B **(E)** were measured using ELISA. Values are reported in pg/ml and represent the mean ± SD of 4 independent experiments, each performed in triplicates. Paired Student’s t-test was used for the statistical analysis. *P<0.001 vs. NK cells; **P<0.001 vs. NK+IL2. L-MSC, liver mesenchymal stromal cell; BM-MSC, bone marrow mesenchymal stromal cell; A-MSC, adipose mesenchymal stromal cell; NK, natural killer.

Secretion of pro-inflammatory cytokines, particularly IFNγ and TNFα, is an important component of the activation-induced signaling that enhances NK cells’ cytolytic activity. Therefore, next, we assessed the impact of MSCs on NK cells’ capacity to produce these two cytokines. Stimulation with IL-2 increased secretion of IFNγ (68.8 ± 7.2 pg/ml) by NK cells (2.4 ± 0.6 pg/ml by unstimulated NK cells; P<0.001). IFNγ secretion by NK cells decreased significantly following co-cultures with MSCs for 3 days. The highest degree of inhibition was observed after exposure to L-MSCs (25.9 ± 2.5 pg/ml), followed by BM-MSCs (32.4 ± 1.9 pg/ml) and A-MSCs (39.4 ± 6.3 pg/ml) (**Figure 5A**). The difference in IFNγ secretion after L-MSC co-cultures vs. either BM-MSC or A-MSC co-cultures was significant (P=0.02 and P<0.001, respectively). A similar pattern was observed in TNFα levels, with increased concentrations after IL-2 stimulation (11.9 ± 1.4 vs. 79.8 ± 7.0 pg/ml, P<0.0001). TNFα levels decreased to 9.5 ± 0.9, 10.4 ± 1.7, 25.1 ± 2.4 after co-cultures with L-MSCs, BM-MSCs, A-MSCs, respectively (P<0.001 for all) (**Figure 5B**). The supernatants from MSC cultures without NK cells were used as negative control, which secreted no IFNγ or TNFα (data not shown here).

### NK cell cytotoxic activity after exposure to MSC

**Figure 6:**
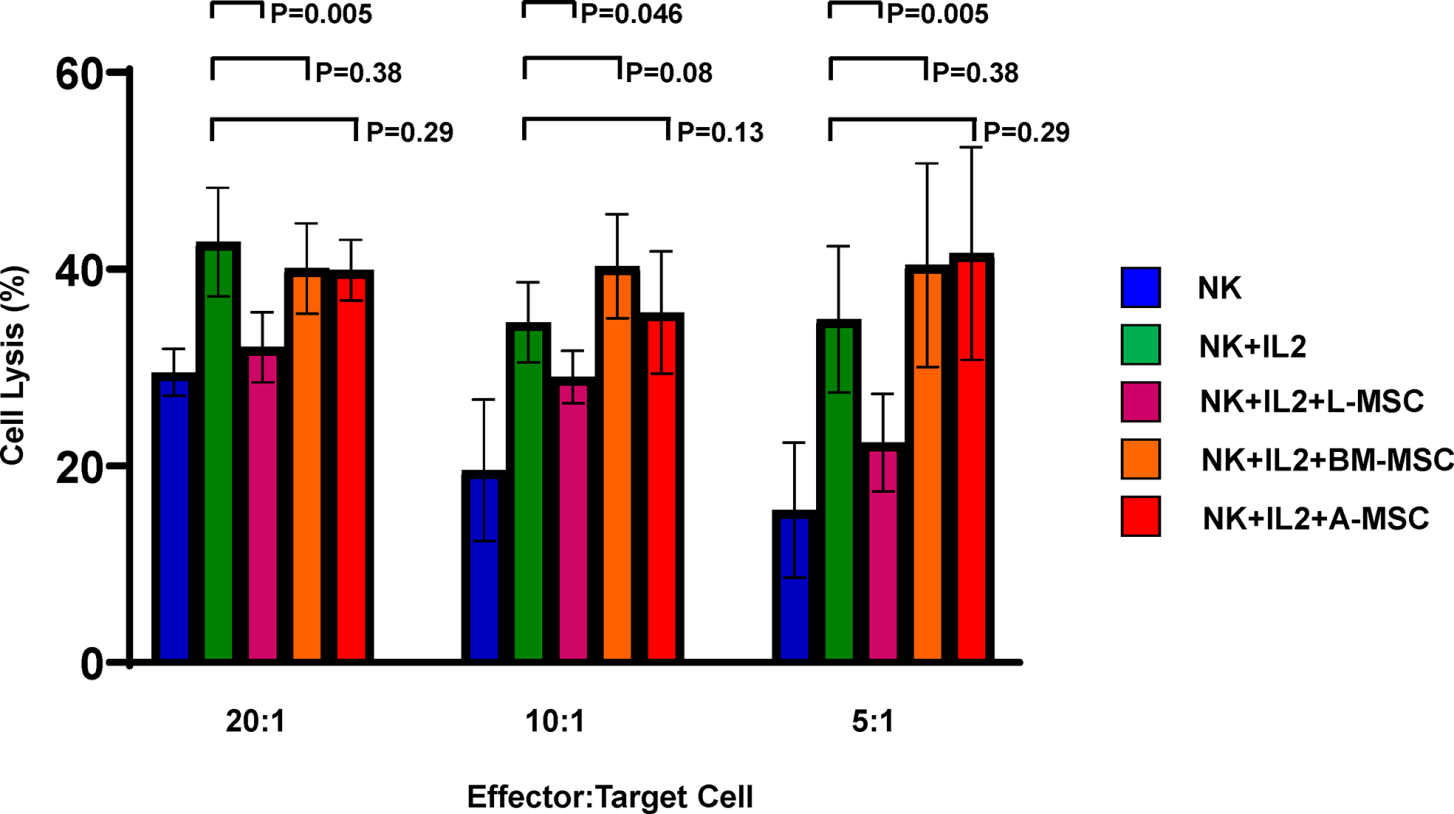
MSCs’ impact on NK cell cytolytic activity. NK cells cultured alone with or without IL-2 or in direct contact with MSCs from different sources for 72 h at 10:1 NK : MSC ratio. NK cells were harvested and used as effector cells in a Cr51 release assay against K562 target cells at different effector/target cell ratios. Data is presented as percent lysis of the target cells and is representative of 6 independent experiments. L-MSC, liver mesenchymal stromal cell; BM-MSC, bone marrow mesenchymal stromal cell; A-MSC, adipose mesenchymal stromal cell; NK, natural killer.

Because exposure to L-MSCs, but not to A-MSCs or BM-MSCs, appeared to decrease the surface expression of the activating receptors on NK cells and each MSC type was found to influence proinflammatory cytokine secretion differently, next, we investigated the cytolytic function of NK cells after exposure to MSC. Freshly isolated NK cells were co-cultured with L-MSCs, BM-MSCs or A-MSCs at 10:1 NK : MSC ratio for 3 days, and their cytolytic activity was assessed by Cr51 release of the labeled K562 tumor line at the end of the culture period. As expected, stimulation with IL-2 of NK cells that were not co-cultured with MSCs increased their cytolytic activity from 29.5% ± 2.4 to 42.8% ± 5.5; 19.6% ± 7.2 to 31.8% ± 7.2, and 15.5% ± 6.8 to 31.6% ± 10.9, at 20:1, 10:1 and 5:1 effector:target ratios, respectively (**Figure 6**). Following co-culture with L-MSCs, NK cells’ cytolytic activity reduced significantly at all effector:target ratios (32.7% ± 3.9 at 20:1, 26.5% ± 5.9 at 10:1 and 24.7% ± 7.8 at 5:1, P<0.05 for all, compared to IL-2-stimulted NK cells). In contrast, exposure to neither BM-MSCs nor A-MSCs altered the cytolytic activity of NK cells.

### Mechanisms of L-MSC-induced reduction in NK cell cytotoxicity

In order to better understand the mechanisms of the L-MSC-induced reduction in NK cytotoxicity, first we tested the cytolytic/cytotoxic proteins in NK supernatants before and after co-cultures with each MSC type. The secreted perforin levels were significantly increased after IL-2 stimulation (1872 ± 86 vs. 3325 ± 381, P<0.0001). After co-cultures with L-MSCs or A-MSCs, the level of perforin did not change (3025.8 ± 240.2 and 3245.9 ± 109.8, respectively). However, perforin levels were decreased following BM-MSC co-cultures significantly (2410 ± 226, P=0.001) (**Figure 5C**). Similarly, the exposure to either L-MSCs or A-MSCs did not affect the concentration of granzyme A or B, whereas BM-MSCs decreased the concentration of both (**Figures 5D, E**). The supernatants from MSC cultures without NK cells were used as the negative control, which secreted no perforin, granzyme A or granzyme B (data not shown).

**Figure 7:**
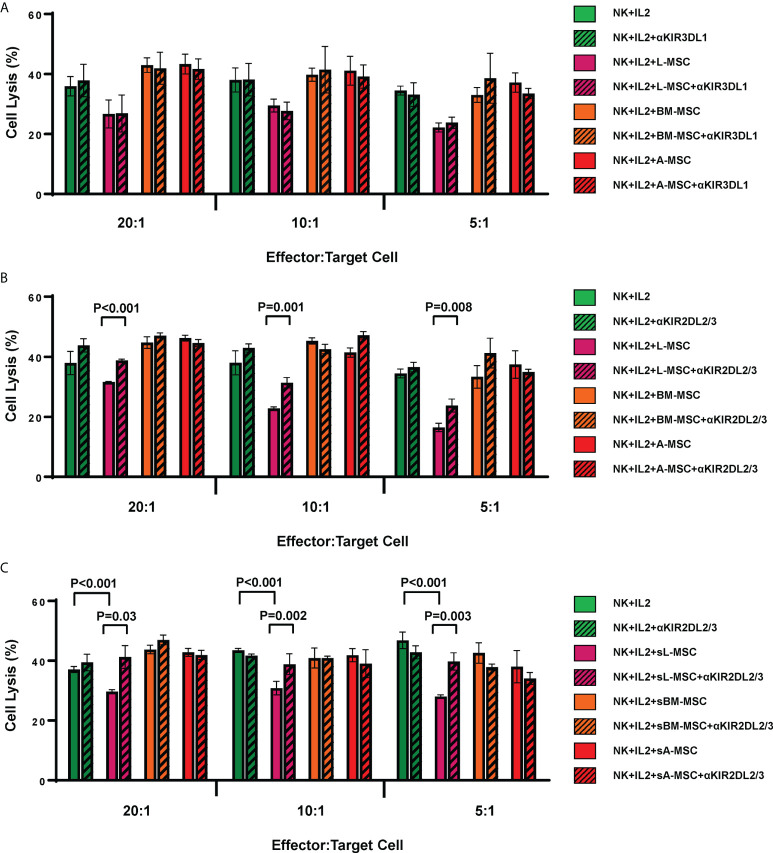
L-MSCs inhibit NK cell cytolytic function through secretion of soluble HLA-C. The two highly expressed NK cell inhibitors in the L-MSC secretome, soluble HLA-B-Bw4 and HLA-C1 were tested for their role in L-MSC-induced inhibition of NK cell cytolysis. The blockade of the KIR3DL1, receptor for HLA-B-Bw4 did not have an impact **(A)**, while blockade of KIR2DL2/3, receptor for HLA-C1 reversed the NK cell inhibition **(B)**. When NK cells were cultured for 3 days with supernatants of L-MSC cultures (sL-MSC), their cytolytic function was inhibited, whereas BM-MSC or A-MSC culture supernatants (sBM-MSC and sA-MSC, respectively) did not impair the NK cytolytic activity **(C)**. The results are representative of 3 independent experiments for panel A&B, and 2 independent experiments for panel **(C)** Each experiment was done in triplicates per groups. Paired Student’s t-test was used for the statistical analysis. L-MSC, liver mesenchymal stromal cell; BM-MSC, bone marrow mesenchymal stromal cell; A-MSC, adipose mesenchymal stromal cell; NK, natural killer.

As the L-MSC-induced decrease in NK cytotoxicity did not appear to be mediated through cytotoxic/cytolytic proteins, we manually curated a list of proteins known to inhibit NK functions and performed an overlap analysis of these with the secretome data. Using a fold-change of >2 in mean intensity with a P value of <0.05, we identified 2 potential mediators of the L-MSC-induced reduction in NK cytotoxicity: HLA-B-Bw4 and HLA-C1. We then repeated the cytotoxicity assays using NK cells co-cultured with different types of MSCs, with and without antibodies blocking KIR3DL1 and KIR2DL2/3, respective receptors for HLA-B-Bw4 and HLA-C1. While blockade of KIR3DL1 did not change the cytolytic activity of NK cells co-cultured with L-MSCs (**Figure 7A** **),** blockade of KIR2DL2/3 reversed the L-MSC-mediated reduction in NK cell cytolysis at all effector:target cell ratios tested (P<0.001, P=0.001 and P=0.08 at 20:1, 10:1 and 5:1, respectively) (**Figure 7B**). The blockade of KIR2DL2/3 restored the NK cells’ cytolytic activity to pre-L-MSC exposure levels at the 20:1 ratio (P<0.001), but the reversal was only partial at 10:1 and 5:1 ratio. To test whether the inhibitory effect of L-MSC on NK cytotoxicity was dependent on cell-to-cell contact, we repeated the assays without co-culturing the NK cells with MSC but exposing them to the MSC culture supernatants for 3 days. NK cells exposed to L-MSC culture supernatant demonstrated reduced cytotoxicity at all effector:target cell ratios (P<0.001 each), whereas exposure to neither the BM-MSC nor the A-MSC supernatants impacted the NK cytotoxicity, suggesting that the mechanisms through which the inhibition occurs are mediated through soluble factors. The blockade of KIR2DL2/3 reversed the L-MSC supernatant-mediated inhibition of the NK cell cytotoxicity (**Figure 7C**). All the MSC cell lines were confirmed to be at least heterozygous for HLA-C1 genotype (**Table 3**).

**Table 3:** HLA-C profiles of each of the MSC lines tested.

	HLA-C alleles					
**L-MSC**	C*07/C*07	C*01/C*05	C*06/C*07	C*07/C*07	C*03/C*17	C*04/C*07
**BM-MSC**	C*03/C*08	C*03/C*16	C*04/C*07			
**A-MSC**	C*07/C*07	C*04/C*12	C*03/C*03			

DNA-based typing was performed utilizing the sequence-specific oligonucleotide (SSO) probes, using LABType (One Lambda, Canoga Park, CA), as described previously (31). Alleles in bold font are those of the HLA-C1 genotype.

## Discussion

The liver is an immunologically active organ with a preponderance towards tolerance at baseline, due to its unique location between the endotoxin-rich portal system and the central venous system. Remarkably, the tolerogenic properties of the liver persist after its transplantation from one individual to another, evidenced by the facts that; 1) the liver transplant recipients are maintained on the lowest doses of immunosuppression among all solid organ transplant recipients; and 2) the liver allografts reduce the incidence of rejection and alloimmune-mediated injury of the simultaneously transplanted other organs from the same donor. Several lines of evidence suggest that the immune cells within the liver are uniquely different than their counterparts elsewhere in the body, and their interaction with the hepatocytes and sinusoidal endothelial cells play crucial roles in creating the tolerogenic microenvironment (34–37). We have hypothesized that liver-resident MSCs also play a role in this unique phenomenon and have been investigating the immunomodulatory properties of L-MSCs in comparison to those of A-MSCs and BM-MSCs.

MSCs are non-hematopoietic multipotent cells found in the perivascular stroma of many tissues and organs. They have the potential to differentiate into cells of mesenchymal origin, and play important roles in tissue repair, regeneration and angiogenesis. They also possess potent immunomodulatory properties. Their impact on regulating lymphocytes, macrophages, dendritic cells and NK cells is widely reported. The tissue source of MSCs influence their immunomodulatory properties, presumably secondary to epigenomic mechanisms (38). We have previously demonstrated that L-MSCs have a distinctly different transcriptome than A-MSCs or BM-MSCs, with upregulated transcripts and gene sets of immune regulation and immunomodulation (26). These differences translate to functional differences, as L-MSCs are superior to A-MSCs and BM-MSCs in their capacity to inhibit alloreactive T cell proliferation.

NK cells are important components of the innate immune system. While they are primarily involved in the immune response against infected cells and tumors, they also contribute to the inflammatory response and rejection after transplantation. Most NK cells have low CD56 (CD56^dim^) expression in the human peripheral blood, whereas approximately 10% of NK cells are CD56^bright^ (39). Their ability to lyse target cells and provide an early source of immunoregulatory cytokines is based on their population, such as the CD56^dim^ population are more cytotoxic (40). In the current study, we analyzed the impact of MSCs from different tissue sources on NK cells. To simulate the biological variances, we elected to use freshly isolated NK cells from healthy donors. As expected, more than 80% of NK cells were CD56^dim^ in our donors. After IL2 stimulation, the CD56^bright^ population slightly increased while decreased following co-cultures with MSCs.

The NK cell cytotoxicity mechanisms are regulated by a diverse set of activating and inhibitory receptors (41). In the current study we found that the surface expression of the natural cytotoxicity (activating) receptors, NKp44 and NKG2D, was reduced in the CD56^dim^ NK cell subset co-cultured with A-MSCs and BM-MSCs, consistent with what has been observed previously (27, 28). However, the surface expression of these receptors was inhibited at a greater level when NK cells were co-cultured with L-MSCs. Among the CD56^bright^ subset, the inhibition was limited only to NKp30, potentially reflecting the fact that the CD56^bright^ subset is primarily recognized for their cytokine secretion rather than cytotoxicity (27, 41). Expression of CD16, another NK cell activating receptor, was not affected after co-cultures with either of the MSCs.

As our observation that L-MSCs were superior inhibitors of the activating receptor expression on the surface of NK cells suggested that they might also impair NK cell cytotoxicity, we tested the cytolytic activity of NK cells on K562 target cells. Indeed, we demonstrate that exposure to L-MSCs resulted in the highest degree of reduction in NK cells’ cytotoxicity. At the same time, NK cells co-cultured with L-MSCs produced the least amount of IFNγ. These findings, while novel, are in parallel to what our group has demonstrated previously on L-MSCs’ impact on alloreactive T cell proliferation. Although the MSC tested from all three sources in our experiments are phenotypically similar, their impact on NK cell cytolytic function and cytokine secretion are uniquely different, with better inhibition achieved by L-MSCs. MSCs isolated from quiescent tissues have been previously reported to have less immunosuppressive features when compared to MSCs from tissues with persistent inflammatory stimuli (42). The liver, with its inflammatory stimuli-rich microenvironment (43), likely accounts for the superior immunosuppressive features of L-MSCs compared to MSCs from other tissue sources.

MSCs exert their immunomodulatory effect through secreted proteins (44). This has been shown both through *in vitro* studies using transwell systems that prevent cell-to-cell contact, as well as through *in vivo* studies that use MSC exosomes, rather than the cells (45). Among the many soluble mediators of MSC-mediated immunomodulation are TGF-β that promotes the differentiation of naïve T cells into Treg cells (46), PGE2 that induces dendritic cells to upregulate the anti-inflammatory cytokine IL-10 while reducing their secretion of pro-inflammatory TNF-α and IL-12 (18, 47), and IL-6 that helps inhibit apoptosis of lymphocytes and assist their survival and differentiation into antibody-secreting cells (48). The profile of the soluble proteins produced by MSCs differs primarily according to their tissue source (49). In the current study, we found that L-MSC secretome is uniquely different than nL-MSC even though the cells were cultured simultaneously and under the same conditions. Regardless of the tissue source, all MSCs shared a secretome reflective of proteins that provide a trophic niche, one of the main features of these cells. The main difference that separated the secretome of L-MSC from that of others was the enrichment of protein groups that typically play roles in cells’ response to inflammatory signals. In contrast, the nL-MSC secretome was enriched with pathways that are important for tissue integrity and repair. Remarkably, the patten of the differences in secretome is similar to the pattern we had previously reported in the MSC transcriptome. As such, the data suggest that the L-MSCs are programmed to respond to inflammatory signals more readily, which leads to upregulation of the mechanisms responsible for their immunomodulatory properties. It should be noted that the L-MSCs were not exposed to any stimuli, such as IFNγ, any time during the culture period. Thus, it is likely that they are pre-exposed to IFNγ in the liver, where the microenvironment is rich in IFNγ, and this is one of the drivers of their naturally more immunomodulatory phenotype. The secretome data also allowed us to identify possible candidates as mediators of the L-MSC-induced reduction in NK cell cytolytic function. L-MSC secretome is enriched with soluble HLA class I molecules, particularly HLA-C. Given that the NK cell cytotoxic functions are inhibited when their cell surface inhibitory receptors interact with HLA class I molecules, we hypothesized that the soluble HLA-C-rich secretome of L-MSCs contribute to the decreased NK cell cytotoxicity. When the receptor for HLA-C was blocked, we indeed found that this observation is, at least partially, reversed. In line with our hypothesis that the impact of L-MSC on NK cell cytolytic functions is mediated through secreted proteins, we found that exposure of NK cells only to MSC culture supernatants (i.e. without any contact with the MSCs) resulted in similar degree of inhibition of NK cell cytotoxicity.

While our findings extend our understanding of the unique immunomodulatory properties of L-MSCs, they also provide more support for the use of these cells as therapeutics in immune system-mediated or inflammatory disorders. MSCs have unique properties that make them promising therapeutic tools to be potentially utilized in many medical areas like regenerative medicine and for the treatment of immune system-mediated disorders (50, 51). Their ability to regulate immune responses is the key benefit of the application of MSCs as therapeutics. Given our previous and current findings regarding L-MSCs’ immunoregulatory properties, a better understanding of L-MSCs’ effect on immune cells will improve cell therapy protocols.

This study has several limitations. First, the MSCs tested here were from 12 different individuals, which may have introduced uncontrolled confounding factors that might influence the results. However, the secretome analyses indicate that the main differences in the protein expression stem from the tissue source of the MSCs rather than from the individuals. Second, the sample sizes are small and different among the three groups. Although we have built a sizeable library of MSCs from all three tissue sources, for the current paper, we elected to use the same samples we had used for the transcriptome analyses so that we could test the similarities and differences between the secretome and transcriptome data, as well as the impact of MSCs on alloreactive T cells and NK cells. The enhanced immunomodulatory profile of L-MSCs was confirmed in both studies, despite the fact that the cells tested in the two studies were cultured a year apart, from different frozen samples. Third, while some mechanistic insight is presented in the current study, more detailed investigation of other possibilities and the role IFNγ plays on the MSC-NK cell interaction remains to be explored. The KIR2DL2/3 receptors are evolutionarily preserved and universally expressed on NK cells (52), however, we did not test whether all the NK cells used in these studies expressed KIR2DL2/3. Lastly, the L-MSCs’ superior immunomodulatory properties will need to be tested *in vivo* in future studies.

In summary, our results demonstrate that the secretome of L-MSCs differs from their counterparts from other tissues, with enhanced expression of proteins that are involved in response to inflammatory stimuli. Consequently, L-MSCs are more potent inhibitors of NK cell functions. These results help explain how L-MSCs may contribute to the tolerogenic liver microenvironment and liver-induced systemic tolerance. They also provide further evidence for the use of L-MSCs as therapeutics for disease processes with underlying inflammatory pathophysiology.

## Data availability statement

The datasets presented in this study can be found in online repositories. The names of the repository/repositories and accession number(s) can be found in the article/**Supplementary Material**.

## Author contributions

FY, EO, NA and TT contributed to the conception and design of the study. FY, EO, NA, WDP, MJH and TT collected the data, contributed to the data analysis, and interpretation. FY wrote the first draft of the manuscript. All authors contributed to the article and approved the submitted version.

## Funding

This study was supported by the American Association for the Study of Liver Diseases (AASLD) Foundation 2017 Career Development Award in Liver Transplantation (TT), and the Mayo Clinic E. Rolland Dickson Research Scholarship in Transplantation (TT).

## Conflict of interest

The authors declare that the research was conducted in the absence of any commercial or financial relationships that could be construed as a potential conflict of interest.

## Publisher’s note

All claims expressed in this article are solely those of the authors and do not necessarily represent those of their affiliated organizations, or those of the publisher, the editors and the reviewers. Any product that may be evaluated in this article, or claim that may be made by its manufacturer, is not guaranteed or endorsed by the publisher.
