# Supplementary material for: Liver mesenchymal stem cells are superior inhibitors of NK cell functions through differences in their secretome compared to other mesenchymal stem cells
Source: Front Immunol. 2022 Sep 21;13:952262. doi: 10.3389/fimmu.2022.952262 (PMC9534521; doi:10.3389/fimmu.2022.952262)
Supplement: Supplementary file 1 [file Table_1.docx]

| **Gene Symbol** | **Gene Name** | **Log2 Fold Change** | **P-value** |
| --- | --- | --- | --- |
| COL4A5 | collagen type IV alpha 5 chain | 35.518 | 4.93E-96 |
| CDH6 | cadherin 6 | 35.474 | 2.03E-95 |
| DYNLT1 | dynein light chain Tctex-type 1 | -27.265 | 9.89E-92 |
| IGFBP3 | insulin like growth factor binding protein 3 | -34.246 | 1.92E-84 |
| ARHGDIB | Rho GDP dissociation inhibitor beta | 30.299 | 1.45E-73 |
| PTGFRN | prostaglandin F2 receptor inhibitor | -33.743 | 5.02E-50 |
| COLEC11 | collectin subfamily member 11 | 28.59 | 2.54E-44 |
| IGFBP5 | insulin like growth factor binding protein 5 | 11.11 | 3.52E-31 |
| COL18A1 | collagen type XVIII alpha 1 chain | 4.722 | 6.44E-31 |
| COLEC12 | collectin subfamily member 12 | -32.029 | 9.50E-27 |
| GPC1 | glypican 1 | 3.693 | 5.07E-25 |
| QSOX1 | quiescin sulfhydryl oxidase 1 | 3.907 | 1.52E-21 |
| MMP10 | matrix metallopeptidase 10 | 23.208 | 1.60E-21 |
| HTRA1 | HtrA serine peptidase 1 | -2.549 | 2.39E-17 |
| TFPI2 | tissue factor pathway inhibitor 2 | 28.371 | 4.71E-16 |
| NID2 | nidogen 2 | 5.607 | 8.25E-16 |
| DPT | dermatopontin | -31.142 | 2.35E-15 |
| CDH11 | cadherin 11 | -4.153 | 2.97E-15 |
| CYCS | cytochrome c, somatic | -3.309 | 5.47E-15 |
| SPRR3 | small proline rich protein 3 | -30.033 | 1.24E-13 |
| CCDC80 | coiled-coil domain containing 80 | 2.481 | 5.85E-13 |
| CD276 | CD276 molecule | -29.883 | 8.06E-13 |
| MANF | mesencephalic astrocyte derived neurotrophic factor | -2.427 | 1.00E-12 |
| MARCKS | myristoylated alanine rich protein kinase C substrate | -3.547 | 6.07E-12 |
| CFD | complement factor D | -27.42 | 6.65E-12 |
| SEMA3C | semaphorin 3C | 26.19 | 7.56E-12 |
| CHI3L1 | chitinase 3 like 1 | -35.307 | 1.94E-11 |
| DKK3 | dickkopf WNT signaling pathway inhibitor 3 | 5.074 | 2.61E-11 |
| S100A16 | S100 calcium binding protein A16 | -4.018 | 2.72E-11 |
| GBP1 | guanylate binding protein 1 | 26.182 | 2.88E-11 |
| GAP43 | growth associated protein 43 | -28.503 | 4.45E-11 |
| BAG2 | BAG cochaperone 2 | -2.53 | 2.14E-10 |
| CHID1 | chitinase domain containing 1 | 28.107 | 3.24E-10 |
| NES | nestin | 6.547 | 6.58E-10 |
| GREM1 | gremlin 1, DAN family BMP antagonist | 5.522 | 1.39E-09 |
| C7 | complement C7 | 29.185 | 5.24E-09 |
| SUMO2 | small ubiquitin like modifier 2 | -2.75 | 8.15E-09 |
| SERPINF1 | serpin family F member 1 | -4.227 | 1.07E-08 |
| ADAMTS12 | ADAM metallopeptidase with thrombospondin type 1 motif 12 | 5.404 | 2.10E-08 |
| OLFML2B | olfactomedin like 2B | -33.451 | 2.34E-08 |
| UBE2L3 | ubiquitin conjugating enzyme E2 L3 | -2.462 | 4.78E-08 |
| ITGBL1 | integrin subunit beta like 1 | -3.414 | 5.85E-08 |
| HBB | hemoglobin subunit beta | -3.218 | 6.91E-08 |
| SNRPD1 | small nuclear ribonucleoprotein D1 polypeptide | 2.221 | 7.22E-08 |
| STOM | stomatin | 2.222 | 1.12E-07 |
| PTPRF | protein tyrosine phosphatase receptor type F | 27.865 | 1.57E-07 |
| FST | follistatin | 3.321 | 2.02E-07 |
| HAPLN1 | hyaluronan and proteoglycan link protein 1 | -31.594 | 2.06E-07 |
| PLTP | phospholipid transfer protein | 3.925 | 2.15E-07 |
| ADAMTSL1 | ADAMTS like 1 | -4.757 | 2.22E-07 |
| CRABP2 | cellular retinoic acid binding protein 2 | -18.77 | 2.23E-07 |
| HEG1 | heart development protein with EGF like domains 1 | 25.376 | 2.62E-07 |
| CDV3 | CDV3 homolog | -3.262 | 2.91E-07 |
| PXDN | peroxidasin | 2.953 | 3.13E-07 |
| KRT34 | keratin 34 | -28.812 | 3.39E-07 |
| CFI | complement factor I | -3.184 | 3.77E-07 |
| PPME1 | protein phosphatase methylesterase 1 | 21.63 | 5.66E-07 |
| ARPC2 | actin related protein 2/3 complex subunit 2 | 1.774 | 6.11E-07 |
| IGFBP6 | insulin like growth factor binding protein 6 | -2.612 | 6.49E-07 |
| FLNB | filamin B | 2.257 | 6.99E-07 |
| STC2 | stanniocalcin 2 | 2.971 | 7.69E-07 |
| S100A6 | S100 calcium binding protein A6 | -2.1 | 8.42E-07 |
| SPTBN1 | spectrin beta, non-erythrocytic 1 | 4.51 | 1.11E-06 |
| IPO9 | importin 9 | 24.122 | 1.16E-06 |
| HGF | hepatocyte growth factor | 26.38 | 1.19E-06 |
| PSMB10 | proteasome 20S subunit beta 10 | 2.437 | 1.31E-06 |
| JPT2 | Jupiter microtubule associated homolog 2 | -20.386 | 1.69E-06 |
| COLEC10 | collectin subfamily member 10 | 22.151 | 1.76E-06 |
| OLFML1 | olfactomedin like 1 | 24.895 | 2.46E-06 |
| AZGP1 | alpha-2-glycoprotein 1, zinc-binding | -2.752 | 2.69E-06 |
| APOH | apolipoprotein H | -1.969 | 2.78E-06 |
| IDI1 | isopentenyl-diphosphate delta isomerase 1 | -0.933 | 2.91E-06 |
| PHPT1 | phosphohistidine phosphatase 1 | -1.301 | 3.05E-06 |
| SEMA7A | semaphorin 7A (John Milton Hagen blood group) | -4.705 | 3.43E-06 |
| COL4A1 | collagen type IV alpha 1 chain | 5.86 | 3.65E-06 |
| FSTL1 | follistatin like 1 | -1.519 | 4.01E-06 |
| GOLM1 | golgi membrane protein 1 | -2.876 | 4.57E-06 |
| KRT18 | keratin 18 | 25.289 | 6.90E-06 |
| CCDC25 | coiled-coil domain containing 25 | -2.463 | 7.81E-06 |
| S100A13 | S100 calcium binding protein A13 | -2.285 | 8.14E-06 |
| HBA1/HBA2 | hemoglobin subunit alpha 2 | -4.137 | 9.12E-06 |
| COL7A1 | collagen type VII alpha 1 chain | 4.82 | 9.70E-06 |
| CARS1 | cysteinyl-tRNA synthetase 1 | 3.563 | 1.07E-05 |
| EIF4A2 | eukaryotic translation initiation factor 4A2 | 20.061 | 1.16E-05 |
| ADD1 | adducin 1 | 20.046 | 0.000013 |
| IGHV5-51 | immunoglobulin heavy variable 5-51 | -3.496 | 1.45E-05 |
| SRPX2 | sushi repeat containing protein X-linked 2 | 1.718 | 1.46E-05 |
| TGFBI | transforming growth factor beta induced | 2.952 | 1.46E-05 |
| PLOD1 | procollagen-lysine,2-oxoglutarate 5-dioxygenase 1 | 1.92 | 1.51E-05 |
| COL4A2 | collagen type IV alpha 2 chain | 4.726 | 1.85E-05 |
| FAM3C | FAM3 metabolism regulating signaling molecule C | -1.67 | 1.93E-05 |
| IGHG1 | immunoglobulin heavy constant gamma 1 (G1m marker) | -1.908 | 1.93E-05 |
| APOA1 | apolipoprotein A1 | -1.856 | 2.27E-05 |
| ACYP1 | acylphosphatase 1 | -8.464 | 2.39E-05 |
| CAPG | capping actin protein, gelsolin like | -10.58 | 2.39E-05 |
| UBA2 | ubiquitin like modifier activating enzyme 2 | 23.728 | 2.63E-05 |
| EXT1 | exostosin glycosyltransferase 1 | 3.879 | 0.000028 |
| LUM | lumican | -2.766 | 3.02E-05 |
| COL14A1 | collagen type XIV alpha 1 chain | -29.254 | 3.09E-05 |
| CFB | complement factor B | 4.602 | 3.32E-05 |
| SEC13 | SEC13 homolog, nuclear pore and COPII coat complex component | 1.768 | 0.000034 |
| C1S | complement C1s | 1.677 | 3.42E-05 |
| RPS28 | ribosomal protein S28 | -2.196 | 0.000036 |
| LGALS3BP | galectin 3 binding protein | 1.062 | 3.63E-05 |
| ILF2 | interleukin enhancer binding factor 2 | 2.194 | 3.64E-05 |
| S100A4 | S100 calcium binding protein A4 | -24.552 | 4.25E-05 |
| DNASE2 | deoxyribonuclease 2, lysosomal | -2.95 | 4.43E-05 |
| SERPINB7 | serpin family B member 7 | -3.932 | 4.69E-05 |
| ITGA1 | integrin subunit alpha 1 | 22.903 | 0.000047 |
| PUF60 | poly(U) binding splicing factor 60 | 17.754 | 5.37E-05 |
| VPS26A | VPS26, retromer complex component A | 0.998 | 6.17E-05 |
| FIBIN | fin bud initiation factor homolog | 18.49 | 6.25E-05 |
| PPBP | pro-platelet basic protein | -2.456 | 6.27E-05 |
| PSMB9 | proteasome 20S subunit beta 9 | 1.824 | 6.63E-05 |
| PSME2 | proteasome activator subunit 2 | 1.877 | 0.000067 |
| TBCA | tubulin folding cofactor A | -1.938 | 8.57E-05 |
| NPTX1 | neuronal pentraxin 1 | 23.683 | 8.91E-05 |
| DCN | decorin | 2.622 | 9.04E-05 |
| GSN | gelsolin | 0.975 | 0.000102 |
| OGN | osteoglycin | -24.3 | 0.000115 |
| EXT2 | exostosin glycosyltransferase 2 | 1.563 | 0.00012 |
| HYI | hydroxypyruvate isomerase (putative) | 5.63 | 0.000125 |
| RHOA | ras homolog family member A | -1.484 | 0.00013 |
| PROCR | protein C receptor | 1.817 | 0.000135 |
| RSU1 | Ras suppressor protein 1 | 1.22 | 0.000141 |
| TUBB3 | tubulin beta 3 class III | -2.056 | 0.000155 |
| KRT72 | keratin 72 | -24.6 | 0.000189 |
| PVR | PVR cell adhesion molecule | 1.939 | 0.000191 |
| COPB2 | COPI coat complex subunit beta 2 | 2.692 | 0.000195 |
| RPS12 | ribosomal protein S12 | -1.804 | 0.000202 |
| ANGPTL2 | angiopoietin like 2 | 3.335 | 0.00021 |
| NMI | N-myc and STAT interactor | 18.923 | 0.000217 |
| CORO1B | coronin 1B | 1.26 | 0.00022 |
| CTSB | cathepsin B | -3.182 | 0.000224 |
| JCHAIN | joining chain of multimeric IgA and IgM | -3.254 | 0.000231 |
| CAPN1 | calpain 1 | 1.921 | 0.000238 |
| PLS3 | plastin 3 | 2.179 | 0.000239 |
| HSPB6 | heat shock protein family B (small) member 6 | 18.049 | 0.000247 |
| AIMP1 | aminoacyl tRNA synthetase complex interacting multifunctional protein 1 | -1.202 | 0.000253 |
| IGHG3 | immunoglobulin heavy constant gamma 3 (G3m marker) | -2.072 | 0.00026 |
| YWHAZ | tyrosine 3-monooxygenase/tryptophan 5-monooxygenase activation protein zeta | 1.077 | 0.000265 |
| IGKC | immunoglobulin kappa constant | -1.495 | 0.000266 |
| NRG1 | neuregulin 1 | -21.176 | 0.000276 |
| VAT1L | vesicle amine transport 1 like | 22.692 | 0.000282 |
| IGLC3 | immunoglobulin lambda constant 3 (Kern-Oz+ marker) | -1.657 | 0.0003 |
| TPT1 | tumor protein, translationally-controlled 1 | -1.373 | 0.000309 |
| MANBA | mannosidase beta | -1.35 | 0.000312 |
| TOR1AIP2 | torsin 1A interacting protein 2 | 16.311 | 0.000326 |
| IGHV1-18 | immunoglobulin heavy variable 1-18 | -2.364 | 0.000338 |
| UBE2V1 | ubiquitin conjugating enzyme E2 V1 | -1.45 | 0.000349 |
| IGKV1D-33 | immunoglobulin kappa variable 1D-33 | -1.976 | 0.000352 |
| ADAM12 | ADAM metallopeptidase domain 12 | -2.155 | 0.000355 |
| CORO1C | coronin 1C | 0.893 | 0.00039 |
| SPARC | secreted protein acidic and cysteine rich | -1.306 | 0.000408 |
| FAM20C | FAM20C golgi associated secretory pathway kinase | -23.523 | 0.000453 |
| DDT | D-dopachrome tautomerase | -1.434 | 0.000462 |
| SEPTIN11 | septin 11 | 2.335 | 0.000464 |
| STMN1 | stathmin 1 | -2.941 | 0.0005 |
| SPTAN1 | spectrin alpha, non-erythrocytic 1 | 3.032 | 0.000502 |
| CPA4 | carboxypeptidase A4 | 1.657 | 0.000539 |
| SDC4 | syndecan 4 | 3.053 | 0.000539 |
| CFL1 | cofilin 1 | -1.482 | 0.000545 |
| IQGAP1 | IQ motif containing GTPase activating protein 1 | 1.574 | 0.000555 |
| HP | haptoglobin | -2.282 | 0.000565 |
| HEBP2 | heme binding protein 2 | -1.012 | 0.000567 |
| HSP90AB1 | heat shock protein 90 alpha family class B member 1 | 2.562 | 0.000584 |
| RARRES2 | retinoic acid receptor responder 2 | 20.419 | 0.000588 |
| AFM | afamin | -2.753 | 0.000651 |
| CSTA | cystatin A | -2.359 | 0.000734 |
| MYH9 | myosin heavy chain 9 | 3.409 | 0.00075 |
| ORM2 | orosomucoid 2 | -2.683 | 0.000751 |
| PGA5 (includes others) | pepsinogen A4 | 21.514 | 0.000755 |
| SIL1 | SIL1 nucleotide exchange factor | 5.978 | 0.000812 |
| IGHG2 | immunoglobulin heavy constant gamma 2 (G2m marker) | -1.885 | 0.000822 |
| APRT | adenine phosphoribosyltransferase | -2.152 | 0.000841 |
| SOD1 | superoxide dismutase 1 | -2.002 | 0.000874 |
| PEBP1 | phosphatidylethanolamine binding protein 1 | -1.25 | 0.000909 |
| ECE1 | endothelin converting enzyme 1 | 21.181 | 0.000938 |
| SARS1 | seryl-tRNA synthetase 1 | 20.697 | 0.000942 |
| HLA-B-Bw4 | major histocompatibility complex, class I, B | 23.309 | 0.000304 |
| SDF4 | stromal cell derived factor 4 | 1.659 | 0.001 |
| CXCL12 | C-X-C motif chemokine ligand 12 | 18.251 | 0.00104 |
| PPP1R7 | protein phosphatase 1 regulatory subunit 7 | 17.211 | 0.00104 |
| HPX | hemopexin | -2.064 | 0.00111 |
| CAMK2D | calcium/calmodulin dependent protein kinase II delta | 15.879 | 0.0012 |
| GLIPR2 | GLI pathogenesis related 2 | -17.077 | 0.0012 |
| CRISPLD2 | cysteine rich secretory protein LCCL domain containing 2 | 6.868 | 0.00121 |
| VCP | valosin containing protein | 1.858 | 0.00124 |
| BASP1 | brain abundant membrane attached signal protein 1 | -5.336 | 0.00127 |
| KRT33B | keratin 33B | -23.916 | 0.00128 |
| IGLV3-21 | immunoglobulin lambda variable 3-21 | -2.058 | 0.0013 |
| C1R | complement C1r | 2.022 | 0.00132 |
| TIMP3 | TIMP metallopeptidase inhibitor 3 | -2.476 | 0.00132 |
| IGHV4-59 | immunoglobulin heavy variable 4-59 | -2.084 | 0.00135 |
| ARPC1B | actin related protein 2/3 complex subunit 1B | -0.909 | 0.00137 |
| LIN7C | lin-7 homolog C, crumbs cell polarity complex component | -16.496 | 0.00138 |
| ALDH2 | aldehyde dehydrogenase 2 family member | 20.012 | 0.00142 |
| MGAT5 | alpha-1,6-mannosylglycoprotein 6-beta-N-acetylglucosaminyltransferase | 18.632 | 0.00144 |
| KRT4 | keratin 4 | -5.803 | 0.00148 |
| COL15A1 | collagen type XV alpha 1 chain | 21.265 | 0.0015 |
| P4HA1 | prolyl 4-hydroxylase subunit alpha 1 | 2.733 | 0.00158 |
| CTH | cystathionine gamma-lyase | -19.563 | 0.00159 |
| CRNN | cornulin | -22.172 | 0.00163 |
| GSTO1 | glutathione S-transferase omega 1 | 1.209 | 0.00166 |
| PTGR1 | prostaglandin reductase 1 | 1.32 | 0.00168 |
| NPEPL1 | aminopeptidase like 1 | -5.087 | 0.00172 |
| FKBP1A | FKBP prolyl isomerase 1A | -1.549 | 0.0018 |
| MTAP | methylthioadenosine phosphorylase | -1.404 | 0.0018 |
| EFHD2 | EF-hand domain family member D2 | -1.533 | 0.00181 |
| APOA4 | apolipoprotein A4 | -1.562 | 0.00183 |
| HNRNPU | heterogeneous nuclear ribonucleoprotein U | 17.701 | 0.0019 |
| IGHV3-7 | immunoglobulin heavy variable 3-7 | -2.828 | 0.00191 |
| KRT71 | keratin 71 | -2.29 | 0.00195 |
| PFDN6 | prefoldin subunit 6 | -19.342 | 0.00205 |
| ISYNA1 | inositol-3-phosphate synthase 1 | 18.904 | 0.00211 |
| SERPINB1 | serpin family B member 1 | 0.914 | 0.00213 |
| GNPDA2 | glucosamine-6-phosphate deaminase 2 | -1.03 | 0.00219 |
| S100A9 | S100 calcium binding protein A9 | -2.956 | 0.00223 |
| UAP1 | UDP-N-acetylglucosamine pyrophosphorylase 1 | -1.491 | 0.00231 |
| HSPG2 | heparan sulfate proteoglycan 2 | -1.115 | 0.00234 |
| SERPINB6 | serpin family B member 6 | 2.497 | 0.00238 |
| UNC5C | unc-5 netrin receptor C | 19.64 | 0.00243 |
| S100A14 | S100 calcium binding protein A14 | -4.989 | 0.00245 |
| UBE2A | ubiquitin conjugating enzyme E2 A | -3.023 | 0.00245 |
| PDLIM1 | PDZ and LIM domain 1 | 16.757 | 0.00249 |
| RBP4 | retinol binding protein 4 | -1.733 | 0.00252 |
| LGMN | legumain | -22.186 | 0.00258 |
| QPCT | glutaminyl-peptide cyclotransferase | -15.619 | 0.00263 |
| SEPTIN7 | septin 7 | 2.027 | 0.00263 |
| ANPEP | alanyl aminopeptidase, membrane | -1.907 | 0.00268 |
| KRT86 | keratin 86 | -21.21 | 0.00269 |
| HSP90B1 | heat shock protein 90 beta family member 1 | 2.419 | 0.00273 |
| DCD | dermcidin | -1.39 | 0.00278 |
| PLAUR | plasminogen activator, urokinase receptor | -19.884 | 0.00281 |
| TFRC | transferrin receptor | 2.241 | 0.0029 |
| PDGFRL | platelet derived growth factor receptor like | 18.49 | 0.00291 |
| PSMB8 | proteasome 20S subunit beta 8 | 1.31 | 0.00294 |
| JUP | junction plakoglobin | -2.964 | 0.00296 |
| NUDC | nuclear distribution C, dynein complex regulator | 1.618 | 0.00302 |
| CAPRIN1 | cell cycle associated protein 1 | -0.906 | 0.00307 |
| ORM1 | orosomucoid 1 | -2.386 | 0.00309 |
| ME1 | malic enzyme 1 | 2.843 | 0.0031 |
| PRKAR2A | protein kinase cAMP-dependent type II regulatory subunit alpha | 14.842 | 0.00313 |
| PRDX6 | peroxiredoxin 6 | 1.771 | 0.00325 |
| NTNG1 | netrin G1 | -18.413 | 0.00329 |
| ALDH7A1 | aldehyde dehydrogenase 7 family member A1 | -1.278 | 0.00331 |
| USP14 | ubiquitin specific peptidase 14 | 2.586 | 0.00343 |
| FABP5 | fatty acid binding protein 5 | -2.022 | 0.00348 |
| L3HYPDH | trans-L-3-hydroxyproline dehydratase | -7.04 | 0.00351 |
| RPL10A | ribosomal protein L10a | 1.605 | 0.00352 |
| ATP6V1B2 | ATPase H+ transporting V1 subunit B2 | 15.577 | 0.00357 |
| ANG | angiogenin | -0.876 | 0.00366 |
| RNH1 | ribonuclease/angiogenin inhibitor 1 | -1.101 | 0.0037 |
| RPL30 | ribosomal protein L30 | -1.374 | 0.00374 |
| HHIPL1 | HHIP like 1 | -19.553 | 0.00378 |
| IGKV3-15 | immunoglobulin kappa variable 3-15 | -1.484 | 0.00378 |
| GP1BA | glycoprotein Ib platelet subunit alpha | -19.273 | 0.00382 |
| MICOS10-NBL1/NBL1 | NBL1, DAN family BMP antagonist | -2.188 | 0.00387 |
| AP2B1 | adaptor related protein complex 2 subunit beta 1 | 1.497 | 0.0039 |
| SFRP1 | secreted frizzled related protein 1 | 19.918 | 0.00395 |
| ITGB5 | integrin subunit beta 5 | -20.947 | 0.004 |
| ITM2B | integral membrane protein 2B | 18.549 | 0.00406 |
| PSMD3 | proteasome 26S subunit, non-ATPase 3 | 14.267 | 0.00412 |
| IGKV3-20 | immunoglobulin kappa variable 3-20 | -1.611 | 0.00417 |
| TNC | tenascin C | -1.959 | 0.00417 |
| FKBP10 | FKBP prolyl isomerase 10 | -1.205 | 0.00423 |
| CNN2 | calponin 2 | -6.325 | 0.00425 |
| AGRN | agrin | 3.042 | 0.00429 |
| ERP44 | endoplasmic reticulum protein 44 | 1.888 | 0.00435 |
| HLA-C | major histocompatibility complex, class I, C | 18.707 | 0.0044 |
| SNRPD3 | small nuclear ribonucleoprotein D3 polypeptide | 1.25 | 0.00441 |
| IL6ST | interleukin 6 signal transducer | 16.886 | 0.00444 |
| C4A/C4B | complement C4A (Rodgers blood group) | 0.993 | 0.00452 |
| S100A8 | S100 calcium binding protein A8 | -2.28 | 0.00453 |
| KRT35 | keratin 35 | -28.312 | 0.00462 |
| HSPA5 | heat shock protein family A (Hsp70) member 5 | 1.055 | 0.00465 |
| DLD | dihydrolipoamide dehydrogenase | -1.31 | 0.00474 |
| FLNC | filamin C | 1.166 | 0.00475 |
| KRT73 | keratin 73 | -19.19 | 0.00484 |
| VPS35 | VPS35 retromer complex component | 18.492 | 0.00484 |
| ENPP1 | ectonucleotide pyrophosphatase/phosphodiesterase 1 | -23.931 | 0.0049 |
| GALNT2 | polypeptide N-acetylgalactosaminyltransferase 2 | 1.28 | 0.0049 |
| RPS20 | ribosomal protein S20 | -1.214 | 0.00492 |
| PSMA3 | proteasome 20S subunit alpha 3 | 1.189 | 0.00493 |
| TIMP4 | TIMP metallopeptidase inhibitor 4 | -9.857 | 0.00496 |
| CTSK | cathepsin K | -17.098 | 0.00497 |
| MFAP2 | microfibril associated protein 2 | -1.45 | 0.005 |
| IGHA2 | immunoglobulin heavy constant alpha 2 (A2m marker) | -2.37 | 0.00501 |
| NID1 | nidogen 1 | 1.906 | 0.00501 |
| LYZ | lysozyme | -1.875 | 0.0051 |
| P4HB | prolyl 4-hydroxylase subunit beta | 1.551 | 0.00516 |
| PSMC1 | proteasome 26S subunit, ATPase 1 | 17.558 | 0.00518 |
| IGHA1 | immunoglobulin heavy constant alpha 1 | -1.762 | 0.0052 |
| DDB1 | damage specific DNA binding protein 1 | 1.224 | 0.00527 |
| EZR | ezrin | 3.198 | 0.00529 |
| PCOLCE2 | procollagen C-endopeptidase enhancer 2 | -19.142 | 0.00535 |
| RAB6A | RAB6A, member RAS oncogene family | -1.509 | 0.00543 |
| APP | amyloid beta precursor protein | 1.924 | 0.00551 |
| LMNA | lamin A/C | 2.18 | 0.00564 |
| SRPRA | SRP receptor subunit alpha | 12.607 | 0.00574 |
| TGM3 | transglutaminase 3 | -2.532 | 0.00581 |
| ARCN1 | archain 1 | 0.821 | 0.00592 |
| PSME1 | proteasome activator subunit 1 | 0.962 | 0.00592 |
| TFPI | tissue factor pathway inhibitor | 18.401 | 0.00599 |
| VCL | vinculin | 1.121 | 0.00599 |
| ADD3 | adducin 3 | 14.264 | 0.0062 |
| IGHV1-69 | immunoglobulin heavy variable 1-69 | -1.504 | 0.00621 |
| IGHG4 | immunoglobulin heavy constant gamma 4 (G4m marker) | -1.763 | 0.00623 |
| NCL | nucleolin | 2.727 | 0.00626 |
| CSRP1 | cysteine and glycine rich protein 1 | -1.344 | 0.0064 |
| BAX | BCL2 associated X, apoptosis regulator | 2.33 | 0.00655 |
| PAMR1 | peptidase domain containing associated with muscle regeneration 1 | 5.863 | 0.00657 |
| DSC1 | desmocollin 1 | -2.599 | 0.00665 |
| F10 | coagulation factor X | 16.902 | 0.00669 |
| TFG | trafficking from ER to golgi regulator | 1.742 | 0.00669 |
| CP | ceruloplasmin | -1.72 | 0.00688 |
| XRCC6 | X-ray repair cross complementing 6 | 14.084 | 0.00689 |
| ASNS | asparagine synthetase (glutamine-hydrolyzing) | 1.963 | 0.00692 |
| MAMDC2 | MAM domain containing 2 | 20.196 | 0.00693 |
| ASS1 | argininosuccinate synthase 1 | -2.276 | 0.0071 |
| HSP90AA1 | heat shock protein 90 alpha family class A member 1 | 2.85 | 0.00717 |
| IGKV4-1 | immunoglobulin kappa variable 4-1 | -1.843 | 0.00718 |
| C1QTNF1 | C1q and TNF related 1 | 19.953 | 0.00727 |
| LOXL3 | lysyl oxidase like 3 | -3.388 | 0.00732 |
| TGFB1 | transforming growth factor beta 1 | -1.23 | 0.00735 |
| JPT1 | Jupiter microtubule associated homolog 1 | -13.154 | 0.0074 |
| BTF3 | basic transcription factor 3 | -16.785 | 0.00744 |
| KCTD12 | potassium channel tetramerization domain containing 12 | -13.941 | 0.00749 |
| MVP | major vault protein | 1.581 | 0.00758 |
| PF4 | platelet factor 4 | -2.061 | 0.00762 |
| ARHGAP1 | Rho GTPase activating protein 1 | 1.489 | 0.00764 |
| KRT31 | keratin 31 | -21.159 | 0.00784 |
| UBLCP1 | ubiquitin like domain containing CTD phosphatase 1 | 16.25 | 0.00793 |
| PLIN3 | perilipin 3 | -1.56 | 0.0081 |
| RNASET2 | ribonuclease T2 | -1.323 | 0.0081 |
| THBS2 | thrombospondin 2 | 1.003 | 0.00825 |
| JAG1 | jagged canonical Notch ligand 1 | 19.019 | 0.00831 |
| MMP19 | matrix metallopeptidase 19 | 19.018 | 0.00833 |
| ANXA10 | annexin A10 | -16.794 | 0.00836 |
| VCAM1 | vascular cell adhesion molecule 1 | 20.853 | 0.00838 |
| QARS1 | glutaminyl-tRNA synthetase 1 | 12.884 | 0.00849 |
| S100A7 | S100 calcium binding protein A7 | -16.399 | 0.00871 |
| PRB1/PRB2 | proline rich protein BstNI subfamily 1 (gene/pseudogene) | -18.36 | 0.00876 |
| ENO1 | enolase 1 | -0.976 | 0.00885 |
| TXNDC5 | thioredoxin domain containing 5 | -0.886 | 0.00885 |
| SERPINB9 | serpin family B member 9 | 18.618 | 0.00886 |
| NHLRC3 | NHL repeat containing 3 | -15.024 | 0.00896 |
| MET | MET proto-oncogene, receptor tyrosine kinase | -19.206 | 0.00897 |
| IGLV3-25 | immunoglobulin lambda variable 3-25 | -1.887 | 0.009 |
| PRPF19 | pre-mRNA processing factor 19 | 17.64 | 0.009 |
| MFAP4 | microfibril associated protein 4 | 3.956 | 0.00907 |
| ABHD14B | abhydrolase domain containing 14B | -1.221 | 0.00914 |
| MACROH2A1 | macroH2A.1 histone | 16.233 | 0.00944 |
| ANXA5 | annexin A5 | 2.024 | 0.00947 |
| KRTAP13-2 | keratin associated protein 13-2 | -18.516 | 0.00951 |
| C1QTNF6 | C1q and TNF related 6 | 16.392 | 0.0096 |
| NDNF | neuron derived neurotrophic factor | -20.48 | 0.00971 |
| ACAA2 | acetyl-CoA acyltransferase 2 | -1.446 | 0.00976 |
| DPYSL3 | dihydropyrimidinase like 3 | 1.78 | 0.01 |
| IGKV2-29 | immunoglobulin kappa variable 2-29 (gene/pseudogene) | -1.73 | 0.0101 |
| MEGF6 | multiple EGF like domains 6 | -16.937 | 0.0101 |
| REXO2 | RNA exonuclease 2 | 0.939 | 0.0101 |
| SMS | spermine synthase | 1.611 | 0.0101 |
| TTR | transthyretin | -2.356 | 0.0101 |
| ELAVL1 | ELAV like RNA binding protein 1 | 1.137 | 0.0108 |
| GC | GC vitamin D binding protein | -1.197 | 0.0109 |
| TF | transferrin | -1.111 | 0.0109 |
| RAB14 | RAB14, member RAS oncogene family | -1.137 | 0.0112 |
| SRC | SRC proto-oncogene, non-receptor tyrosine kinase | -6.974 | 0.0112 |
| ATP6V1A | ATPase H+ transporting V1 subunit A | 17.537 | 0.0114 |
| IGLV3-19 | immunoglobulin lambda variable 3-19 | -1.826 | 0.0114 |
| NXN | nucleoredoxin | 16.328 | 0.0114 |
| CLU | clusterin | -1.338 | 0.0116 |
| NEGR1 | neuronal growth regulator 1 | -5.531 | 0.0116 |
| UGDH | UDP-glucose 6-dehydrogenase | -1.442 | 0.0116 |
| RPS21 | ribosomal protein S21 | -7.306 | 0.0118 |
| YWHAB | tyrosine 3-monooxygenase/tryptophan 5-monooxygenase activation protein beta | 1.188 | 0.0119 |
| P3H1 | prolyl 3-hydroxylase 1 | 1.617 | 0.0122 |
| IGLL1/IGLL5 | immunoglobulin lambda like polypeptide 1 | -1.195 | 0.0123 |
| OTUB1 | OTU deubiquitinase, ubiquitin aldehyde binding 1 | 0.852 | 0.0124 |
| RANBP1 | RAN binding protein 1 | -12.016 | 0.0124 |
| CCL5 | C-C motif chemokine ligand 5 | -1.446 | 0.0125 |
| EDF1 | endothelial differentiation related factor 1 | -1.148 | 0.0126 |
| LRRC17 | leucine rich repeat containing 17 | 13.218 | 0.0126 |
| HNRNPA3 | heterogeneous nuclear ribonucleoprotein A3 | 14.557 | 0.013 |
| HSPB11 | heat shock protein family B (small) member 11 | -1.387 | 0.013 |
| RAB7A | RAB7A, member RAS oncogene family | -0.782 | 0.013 |
| TP53I3 | tumor protein p53 inducible protein 3 | -1.36 | 0.013 |
| DSTN | destrin, actin depolymerizing factor | -1.163 | 0.0132 |
| A1BG | alpha-1-B glycoprotein | -2.408 | 0.0133 |
| SOD3 | superoxide dismutase 3 | 13.028 | 0.0135 |
| HNRNPA1 | heterogeneous nuclear ribonucleoprotein A1 | 2.033 | 0.0136 |
| PLAT | plasminogen activator, tissue type | 15.848 | 0.0136 |
| MAT2A | methionine adenosyltransferase 2A | 1.211 | 0.0137 |
| PAFAH1B2 | platelet activating factor acetylhydrolase 1b catalytic subunit 2 | -0.657 | 0.0137 |
| LAMA5 | laminin subunit alpha 5 | 12.718 | 0.0138 |
| OS9 | OS9 endoplasmic reticulum lectin | 15.951 | 0.0141 |
| BLVRA | biliverdin reductase A | 2.252 | 0.0142 |
| HAPLN3 | hyaluronan and proteoglycan link protein 3 | -21.845 | 0.0142 |
| NIBAN2 | niban apoptosis regulator 2 | 1.25 | 0.0145 |
| IGHV3-15 | immunoglobulin heavy variable 3-15 | -13.067 | 0.0147 |
| KRT85 | keratin 85 | -8.404 | 0.0149 |
| RBP1 | retinol binding protein 1 | 18.289 | 0.015 |
| SEPTIN2 | septin 2 | 4.292 | 0.015 |
| KPNB1 | karyopherin subunit beta 1 | 2.65 | 0.0151 |
| SELENOM | selenoprotein M | -11.433 | 0.0153 |
| ZG16B | zymogen granule protein 16B | -14.818 | 0.0156 |
| H6PD | hexose-6-phosphate dehydrogenase/glucose 1-dehydrogenase | -16.517 | 0.0157 |
| MTHFD1 | methylenetetrahydrofolate dehydrogenase, cyclohydrolase and formyltetrahydrofolate synthetase 1 | -12.913 | 0.0161 |
| S100A10 | S100 calcium binding protein A10 | -1.384 | 0.0163 |
| SFPQ | splicing factor proline and glutamine rich | 13.772 | 0.0163 |
| RPS14 | ribosomal protein S14 | -1.603 | 0.0166 |
| TNFRSF11B | TNF receptor superfamily member 11b | 18.149 | 0.0168 |
| ACO1 | aconitase 1 | 15.853 | 0.0171 |
| ISLR | immunoglobulin superfamily containing leucine rich repeat | -3.267 | 0.0172 |
| A2M | alpha-2-macroglobulin | -1.316 | 0.0173 |
| F3 | coagulation factor III, tissue factor | 17.568 | 0.0173 |
| IGFBP7 | insulin like growth factor binding protein 7 | 1.302 | 0.0174 |
| BOLA2/BOLA2B | bolA family member 2 | -0.654 | 0.0179 |
| VEGFA | vascular endothelial growth factor A | -5.922 | 0.0181 |
| COL12A1 | collagen type XII alpha 1 chain | 1.529 | 0.0182 |
| SRI | sorcin | -15.012 | 0.0182 |
| CD9 | CD9 molecule | 1.71 | 0.0183 |
| RPL7A | ribosomal protein L7a | 15.103 | 0.0186 |
| RTN4 | reticulon 4 | 2.037 | 0.0186 |
| SRP9 | signal recognition particle 9 | -1.765 | 0.0186 |
| VCAN | versican | -1.494 | 0.0186 |
| EIF3A | eukaryotic translation initiation factor 3 subunit A | 14.924 | 0.0187 |
| ANXA7 | annexin A7 | -1.361 | 0.0188 |
| QDPR | quinoid dihydropteridine reductase | -0.81 | 0.0189 |
| PSMA7 | proteasome 20S subunit alpha 7 | 0.612 | 0.019 |
| PRNP | prion protein | -1.347 | 0.0193 |
| SELENOF | selenoprotein F | 12.266 | 0.0193 |
| UBA3 | ubiquitin like modifier activating enzyme 3 | 1.198 | 0.0193 |
| PDCD5 | programmed cell death 5 | -5.968 | 0.0194 |
| MAT2B | methionine adenosyltransferase 2B | 15.638 | 0.0195 |
| PODN | podocan | 12.48 | 0.0195 |
| PAPPA | pappalysin 1 | 12.652 | 0.0197 |
| ACTR3 | actin related protein 3 | 0.673 | 0.0198 |
| TXNRD1 | thioredoxin reductase 1 | 1.239 | 0.0198 |
| KRT13 | keratin 13 | -14.591 | 0.02 |
| HNRNPL | heterogeneous nuclear ribonucleoprotein L | 15.023 | 0.0202 |
| LRG1 | leucine rich alpha-2-glycoprotein 1 | -5.182 | 0.0204 |
| MSN | moesin | 1.733 | 0.0205 |
| GDF15 | growth differentiation factor 15 | 12.776 | 0.0208 |
| AP1M1 | adaptor related protein complex 1 subunit mu 1 | 17.647 | 0.0209 |
| ROBO1 | roundabout guidance receptor 1 | 12.711 | 0.0209 |
| SERPINA3 | serpin family A member 3 | -1.076 | 0.0215 |
| GPS1 | G protein pathway suppressor 1 | 12.942 | 0.0221 |
| SRGN | serglycin | 16.46 | 0.0221 |
| COL11A1 | collagen type XI alpha 1 chain | -17.639 | 0.0223 |
| DPP4 | dipeptidyl peptidase 4 | 16.921 | 0.0227 |
| COPB1 | COPI coat complex subunit beta 1 | 15.536 | 0.0228 |
| SF3B3 | splicing factor 3b subunit 3 | 12.235 | 0.0229 |
| NUTF2 | nuclear transport factor 2 | -1.02 | 0.023 |
| PAM | peptidylglycine alpha-amidating monooxygenase | 2.402 | 0.0233 |
| PTRHD1 | peptidyl-tRNA hydrolase domain containing 1 | 10.826 | 0.0233 |
| INHBA | inhibin subunit beta A | 1.608 | 0.0237 |
| APOA2 | apolipoprotein A2 | -1.366 | 0.0241 |
| CSTB | cystatin B | -0.969 | 0.0242 |
| MMRN1 | multimerin 1 | -0.991 | 0.0243 |
| G6PD | glucose-6-phosphate dehydrogenase | 1.344 | 0.0245 |
| PIN4 | peptidylprolyl cis/trans isomerase, NIMA-interacting 4 | -4.951 | 0.0245 |
| PPL | periplakin | 16.312 | 0.0245 |
| ITIH2 | inter-alpha-trypsin inhibitor heavy chain 2 | 5.074 | 0.0246 |
| IFI35 | interferon induced protein 35 | 12.969 | 0.0248 |
| CFL2 | cofilin 2 | -1.481 | 0.0249 |
| ADAM9 | ADAM metallopeptidase domain 9 | 0.951 | 0.0251 |
| CDH2 | cadherin 2 | -0.814 | 0.0252 |
| CA2 | carbonic anhydrase 2 | -12.888 | 0.0257 |
| KRT81 | keratin 81 | -15.331 | 0.0259 |
| HNRNPA2B1 | heterogeneous nuclear ribonucleoprotein A2/B1 | 1.096 | 0.0261 |
| PYGB | glycogen phosphorylase B | 1.959 | 0.0261 |
| ENO3 | enolase 3 | -15.651 | 0.0262 |
| PAK2 | p21 (RAC1) activated kinase 2 | 10.135 | 0.0262 |
| ETF1 | eukaryotic translation termination factor 1 | 2.071 | 0.0265 |
| FHL2 | four and a half LIM domains 2 | 1.684 | 0.0266 |
| FBN2 | fibrillin 2 | -16.865 | 0.0268 |
| GATD3A/GATD3B | glutamine amidotransferase like class 1 domain containing 3A | 11.498 | 0.0268 |
| OLA1 | Obg like ATPase 1 | 15.876 | 0.0268 |
| YWHAE | tyrosine 3-monooxygenase/tryptophan 5-monooxygenase activation protein epsilon | 0.949 | 0.0271 |
| SCARB2 | scavenger receptor class B member 2 | -18.575 | 0.0272 |
| TPD52L2 | TPD52 like 2 | -3.747 | 0.0272 |
| AGA | aspartylglucosaminidase | -1.836 | 0.0274 |
| PPIA | peptidylprolyl isomerase A | -0.98 | 0.0275 |
| IPO5 | importin 5 | 16.36 | 0.0276 |
| CSPG4 | chondroitin sulfate proteoglycan 4 | 10.542 | 0.0277 |
| SSB | small RNA binding exonuclease protection factor La | 12.123 | 0.0281 |
| DCTN2 | dynactin subunit 2 | -1.351 | 0.0282 |
| PREPL | prolyl endopeptidase like | 13.075 | 0.0283 |
| PRKAR1A | protein kinase cAMP-dependent type I regulatory subunit alpha | 1.293 | 0.0291 |
| MAN2B1 | mannosidase alpha class 2B member 1 | -5.088 | 0.0302 |
| GNG12 | G protein subunit gamma 12 | -1.399 | 0.0303 |
| OSCAR | osteoclast associated Ig-like receptor | 11.885 | 0.0305 |
| EML2 | EMAP like 2 | 6.633 | 0.0308 |
| ADAM10 | ADAM metallopeptidase domain 10 | 1.142 | 0.031 |
| NAP1L1 | nucleosome assembly protein 1 like 1 | 15.436 | 0.031 |
| YWHAH | tyrosine 3-monooxygenase/tryptophan 5-monooxygenase activation protein eta | 1.143 | 0.031 |
| DDX39B | DExD-box helicase 39B | 3.858 | 0.0311 |
| S100A11 | S100 calcium binding protein A11 | -1.32 | 0.0312 |
| PTPA | protein phosphatase 2 phosphatase activator | 0.964 | 0.0315 |
| TTYH3 | tweety family member 3 | 12.754 | 0.0315 |
| RO60 | Ro60, Y RNA binding protein | 1.352 | 0.0317 |
| EPPK1 | epiplakin 1 | -4.633 | 0.0318 |
| DNAJC3 | DnaJ heat shock protein family (Hsp40) member C3 | 16.108 | 0.0321 |
| MXRA5 | matrix remodeling associated 5 | -1.573 | 0.0326 |
| MAP4 | microtubule associated protein 4 | 1.28 | 0.0328 |
| ACTN4 | actinin alpha 4 | 1.268 | 0.0329 |
| STC1 | stanniocalcin 1 | 13.969 | 0.0331 |
| FMOD | fibromodulin | 13.922 | 0.0332 |
| KIF23 | kinesin family member 23 | -19.675 | 0.0332 |
| KRTAP2-2 | keratin associated protein 2-2 | -19.237 | 0.0334 |
| ARPC5 | actin related protein 2/3 complex subunit 5 | -1.663 | 0.0336 |
| CCN2 | cellular communication network factor 2 | -1.529 | 0.0336 |
| GNB1 | G protein subunit beta 1 | -1.395 | 0.0341 |
| LRRC15 | leucine rich repeat containing 15 | -18.827 | 0.0341 |
| PRSS23 | serine protease 23 | 2.029 | 0.0341 |
| UTRN | utrophin | -9.805 | 0.0343 |
| VAT1 | vesicle amine transport 1 | -1.549 | 0.0343 |
| CNDP2 | carnosine dipeptidase 2 | 2.627 | 0.0351 |
| EEF2 | eukaryotic translation elongation factor 2 | 1.986 | 0.0356 |
| RAB1B | RAB1B, member RAS oncogene family | -2.456 | 0.0356 |
| GRPEL1 | GrpE like 1, mitochondrial | -1.273 | 0.0357 |
| COL5A2 | collagen type V alpha 2 chain | 1.544 | 0.0361 |
| FNDC1 | fibronectin type III domain containing 1 | -12.509 | 0.0363 |
| ITGA3 | integrin subunit alpha 3 | 13.645 | 0.0363 |
| PPP2R2A | protein phosphatase 2 regulatory subunit Balpha | 13.866 | 0.0363 |
| RARRES1 | retinoic acid receptor responder 1 | -3.144 | 0.0367 |
| CMBL | carboxymethylenebutenolidase homolog | -4.497 | 0.0374 |
| PSMB5 | proteasome 20S subunit beta 5 | -0.421 | 0.0378 |
| IGHV3-74 | immunoglobulin heavy variable 3-74 | -12.811 | 0.0381 |
| MCAM | melanoma cell adhesion molecule | 14.678 | 0.0381 |
| EGFR | epidermal growth factor receptor | 14.382 | 0.0382 |
| HK1 | hexokinase 1 | 13.891 | 0.0383 |
| FCN3 | ficolin 3 | -13.983 | 0.0385 |
| NUDT1 | nudix hydrolase 1 | -1.312 | 0.0386 |
| LAMB2 | laminin subunit beta 2 | 2.368 | 0.0387 |
| POF1B | POF1B actin binding protein | -14.56 | 0.039 |
| GARS1 | glycyl-tRNA synthetase 1 | 0.79 | 0.0394 |
| THY1 | Thy-1 cell surface antigen | -1.348 | 0.0394 |
| ILF3 | interleukin enhancer binding factor 3 | 9.788 | 0.0395 |
| CCL2 | C-C motif chemokine ligand 2 | 2.824 | 0.0399 |
| SF3B4 | splicing factor 3b subunit 4 | 12.788 | 0.04 |
| CA12 | carbonic anhydrase 12 | -17.45 | 0.0401 |
| RPL27A | ribosomal protein L27a | -4.063 | 0.0409 |
| SDF2L1 | stromal cell derived factor 2 like 1 | -1.756 | 0.0409 |
| PTN | pleiotrophin | 13.856 | 0.0412 |
| MOB1A | MOB kinase activator 1A | 11.612 | 0.0421 |
| EIF6 | eukaryotic translation initiation factor 6 | 0.796 | 0.0425 |
| SPRR2A | small proline rich protein 2A | -3.325 | 0.0426 |
| PPM1F | protein phosphatase, Mg2+/Mn2+ dependent 1F | 14.656 | 0.0427 |
| CCDC58 | coiled-coil domain containing 58 | -7.941 | 0.0429 |
| KRT5 | keratin 5 | -1.015 | 0.043 |
| SLC39A14 | solute carrier family 39 member 14 | -14.151 | 0.043 |
| EFEMP2 | EGF containing fibulin extracellular matrix protein 2 | 0.872 | 0.0431 |
| RDX | radixin | 13.184 | 0.0431 |
| KPNA6 | karyopherin subunit alpha 6 | 9.15 | 0.0432 |
| DCTN1 | dynactin subunit 1 | -3.184 | 0.0434 |
| RPS27L | ribosomal protein S27 like | -3.324 | 0.0434 |
| ENPP2 | ectonucleotide pyrophosphatase/phosphodiesterase 2 | -12.267 | 0.0436 |
| RPL11 | ribosomal protein L11 | -1.033 | 0.0444 |
| WDR1 | WD repeat domain 1 | 0.673 | 0.0444 |
| KRT36 | keratin 36 | -14.786 | 0.0446 |
| HDGF | heparin binding growth factor | 1.393 | 0.045 |
| SF3A1 | splicing factor 3a subunit 1 | 10.55 | 0.045 |
| ACTR2 | actin related protein 2 | 0.36 | 0.0451 |
| C5 | complement C5 | -16.242 | 0.0455 |
| UFD1 | ubiquitin recognition factor in ER associated degradation 1 | 10.039 | 0.0455 |
| CALM1 (includes others) | calmodulin 1 | -1.731 | 0.046 |
| EIF4A1 | eukaryotic translation initiation factor 4A1 | 2.244 | 0.0462 |
| IGFBP2 | insulin like growth factor binding protein 2 | -16.16 | 0.0462 |
| SRP19 | signal recognition particle 19 | -12.336 | 0.0469 |
| DPYSL2 | dihydropyrimidinase like 2 | 1.232 | 0.047 |
| CEMIP | cell migration inducing hyaluronidase 1 | 12.343 | 0.0482 |
| ASF1A | anti-silencing function 1A histone chaperone | 14.3 | 0.0483 |
| IL11 | interleukin 11 | 12.197 | 0.0485 |
| CASP14 | caspase 14 | -1.455 | 0.0486 |
| PFDN2 | prefoldin subunit 2 | -9.8 | 0.0486 |
| PITPNB | phosphatidylinositol transfer protein beta | 13.045 | 0.049 |
| DCPS | decapping enzyme, scavenger | 12.79 | 0.0491 |
| NAE1 | NEDD8 activating enzyme E1 subunit 1 | 11.756 | 0.0496 |
| COTL1 | coactosin like F-actin binding protein 1 | -0.726 | 0.0498 |
| SERPINA5 | serpin family A member 5 | -1.306 | 0.0499 |
