# Supplementary figures and images for: Liver mesenchymal stem cells are superior inhibitors of NK cell functions through differences in their secretome compared to other mesenchymal stem cells
Source: Front Immunol. 2022 Sep 21;13:952262. doi: 10.3389/fimmu.2022.952262 (PMC9534521; doi:10.3389/fimmu.2022.952262)

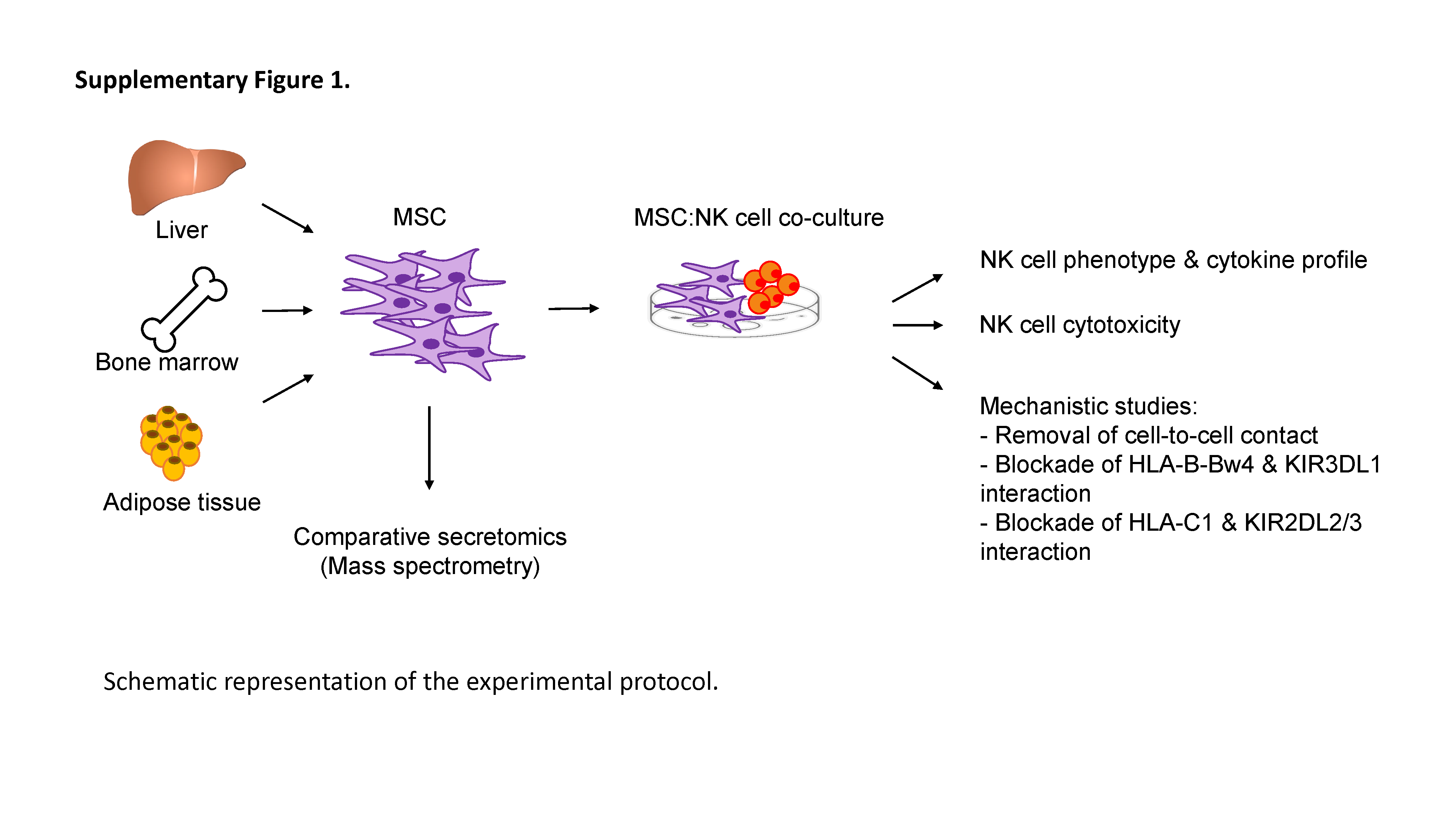

Supplement: Supplementary file 2 [file Image_1.tiff]

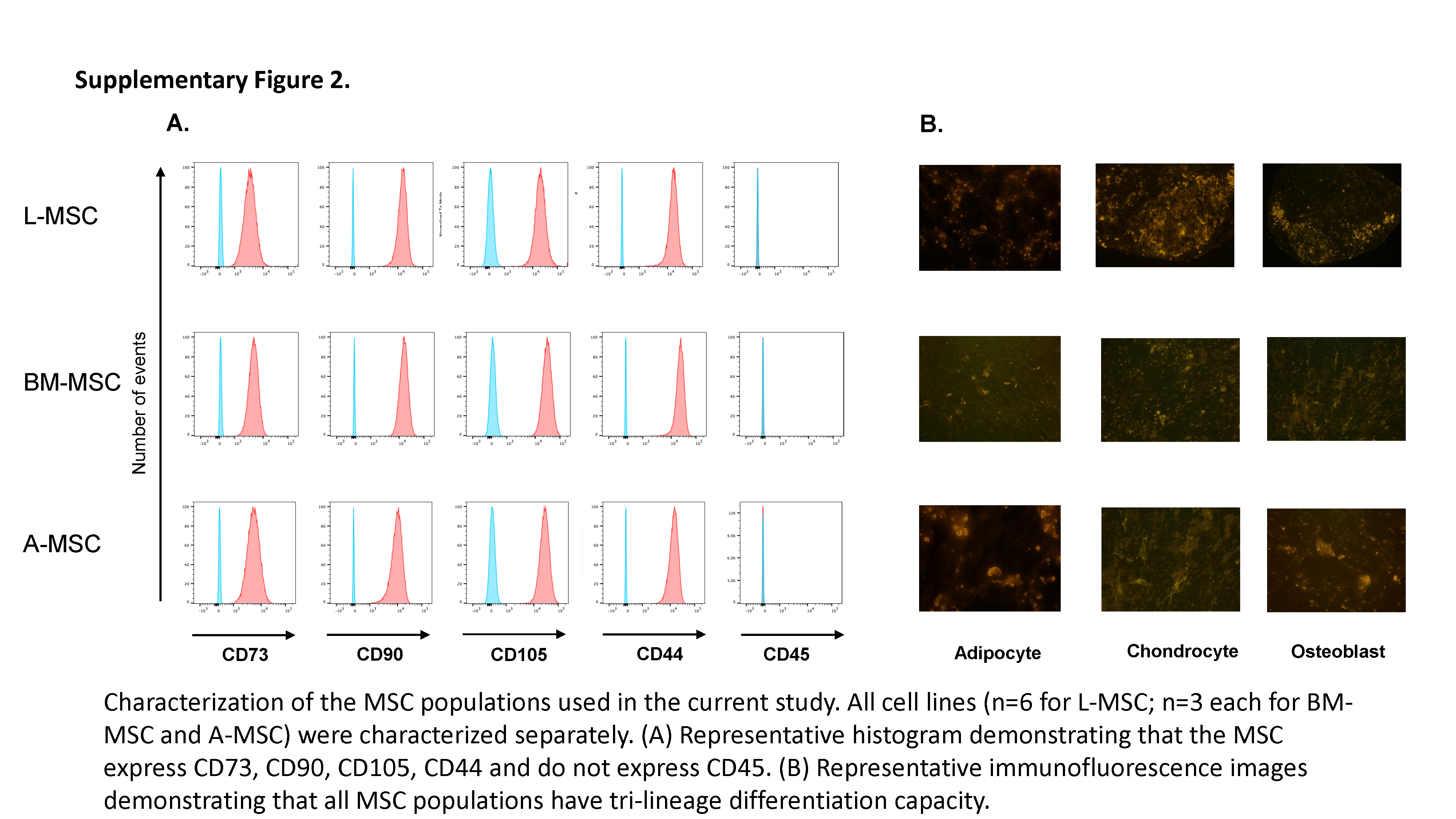

Supplement: Supplementary file 3 [file Image_2.tiff]

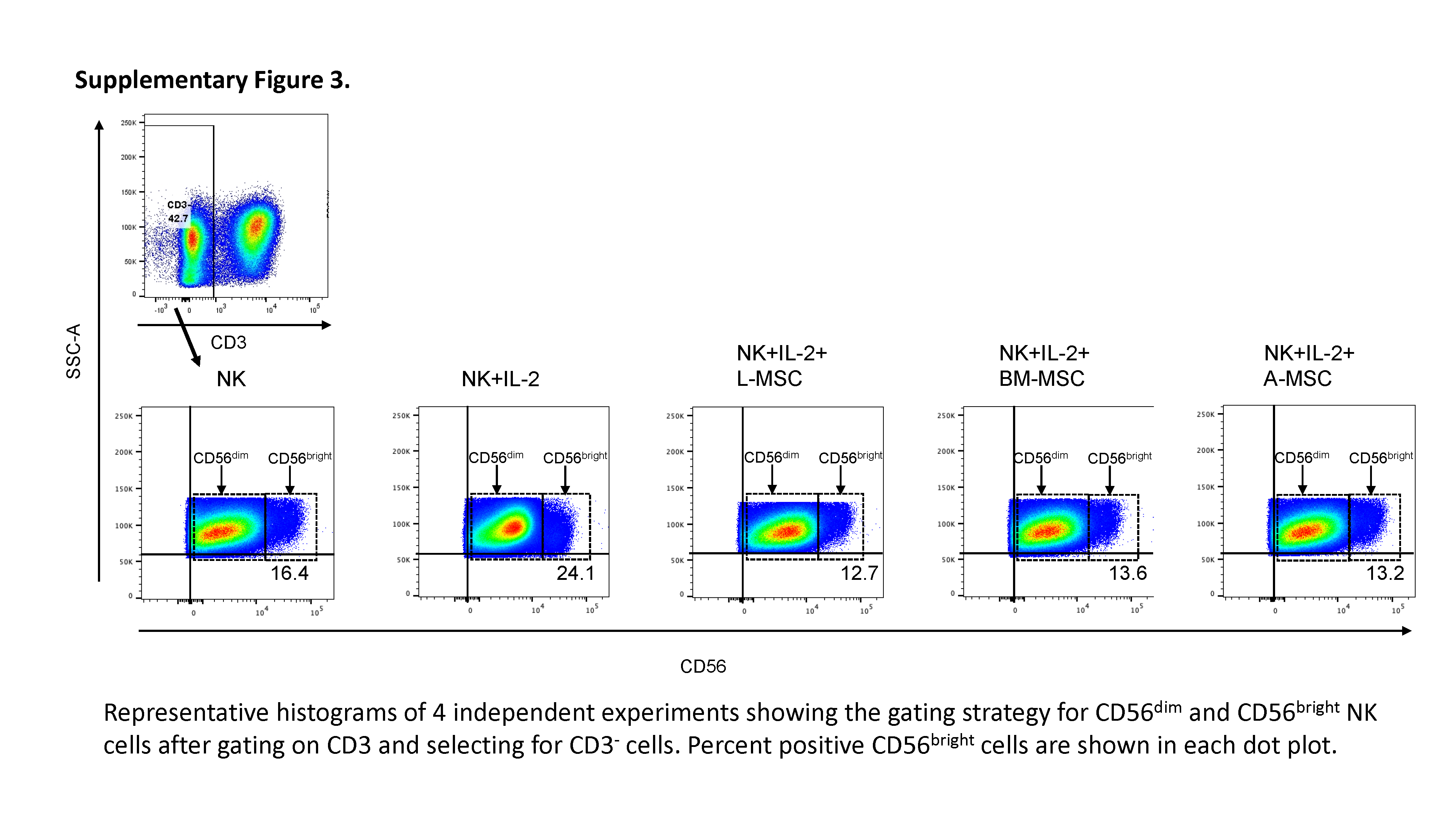

Supplement: Supplementary file 4 [file Image_3.tiff]
